# Enhanced Quenching in an Azaphthalocyanine–Ferrocene Supramolecular Dyad upon Charge-Transfer Complex Formation

**DOI:** 10.1021/acs.inorgchem.5c03733

**Published:** 2025-12-15

**Authors:** Jana Lapesova, Jiri Demuth, Veronika Novakova, Lucie Ludvikova, Tomas Slanina, Petr Zimcik

**Affiliations:** † Department of Pharmaceutical Chemistry and Pharmaceutical Analysis, Faculty of Pharmacy in Hradec Kralove, 37740 Charles University, Akademika Heyrovskeho 1203 50005 Hradec Kralove, Czech Republic; ‡ Institute of Organic Chemistry and Biochemistry of the Czech Academy of Sciences, Flemingovo Namesti 542/2 160 00 Prague 6, Czech Republic

## Abstract

Azaphthalocyanines are fluorescent dyes and photosensitizers with promising applications in photodynamic therapy (PDT) and fluorescence sensing. However, achieving precise control over their photophysical behavior remains a major challenge. Here, we report a supramolecular approach to enhance fluorescence quenching via charge-transfer complex formation. An electron-deficient azaphthalocyanine derivative incorporating a naphthalene-2,6-diol moiety as a charge-transfer donor was synthesized, and its fluorescence response toward a tailored quencher was evaluated. A ferrocene–methylviologen conjugate that simultaneously functions as a quencher and an acceptor in charge-transfer complexes was designed and synthesized for this purpose. Compared to ferrocenemethanol and methylviologen alone, the conjugate quencher exhibited an enhanced quenching efficiency in acetonitrile. The quenching followed a nonlinear Stern–Volmer dependence, indicating both static and dynamic quenching mechanisms, with the former one being more efficient with *K*_S_ = 241 M^–1^ due to directed charge-transfer complex formation between methylviologen and the naphthalene-2,6-diol moiety serving as a staple. These findings demonstrate that complexation can enhance the fluorescence quenching of AzaPc derivatives and suggest a general approach for designing responsive photosensitizers in smart PDT systems or molecular sensing.

## Graphical abstract

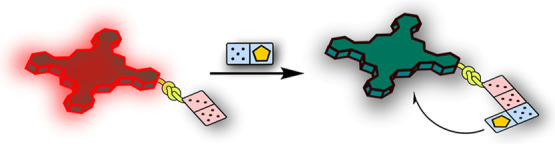

## Introduction

Azaphthalocyanines (AzaPcs) are synthetic dyes formally derived from phthalocyanines (Pcs) that can be used as both photosensitizers in photodynamic therapy (PDT) of cancer and other diseases and theranostic tools or fluorescence sensors.^1^ In all these applications, efficient singlet oxygen production (for PDT) or bright fluorescence (in fluorescence sensors) are advantageous. Recent developments in photosensitizers for PDT are largely focused on the development of “smart” compounds specifically activatable by various stimuli.^2^ For this purpose, the original photoactive state is quenched by various principles, e.g., aggregation,^3^ conjugation with FRET quencher,^4^ or presence of an electron donor.^5^ The last-mentioned principle typically employs the presence of strong donors (e.g., amines^6,7^ or ferrocene^8,9^ ), leading to the quenching of the excited states by electron transfer to the Pc core (acceptor). The quencher is typically attached covalently to the macrocycle and either deactivated after the change of the environment (e.g., protonation in acidic pH)^10^ or removed by the cleavage of a specific labile bond (e.g., disulfide or hydrazone).^9^ Disadvantages of employing the covalent bond are the permanent attachment of the quencher to the fluorophore in the first-mentioned case and the irreversibility of the cleavage in the second one. An alternative mechanism is a change in supramolecular assembly, where various types of noncovalent interactions can be utilized.^11^ As examples, different quenchers have been attached to structurally similar porphyrins and related compounds by axial coordination,^12,13^ hydrogen bonding,^14^ crown ether complexation,^15^ cyclodextrin complexation,^16^ or rotaxane formation.^17^

In this study, we attempted to prove that noncovalent association within charge-transfer complexes (CT-complexes) can also be utilized to extend the pool of available supramolecular quencher attachment methods. Formation of CT-complexes is based on the π–π* interaction of electron-rich (donor) and electron-poor (acceptor) aromatic compounds. Reportedly, CT-complexes have been successfully applied for building self-assembled systems for catalysis^18^ or formation of mechanical bonds in, e.g., catenanes.^19,20^ Their ambipolar electrochemical properties can enhance the conductivity of semiconductors^21^ or the power conversion efficiency of solar cells.^22^ The acceptors of efficient CT-complexes are typically derived from 4,4′-bipyridyl by formal (and mostly also synthetic) alkylation of its nitrogen atoms, producing viologen derivatives, e.g., dimethylviologen (*N*,*N*′-dimethyl-4,4′-bipyridinium). As an example of organic donor moieties, literature reports the use of compounds derived from naphthalene-2,6-diol,^23^ naphthalene-1,5-diol,^24^ hydroquinone,^25^ or tetrathiafulvalene.^26^

Herein, a hypothesis was tested of whether the formation of a supramolecular CT-complex may improve the quenching of the excited state of an AzaPc derivative by ferrocene (Figure 1). Quenching of Pcs by covalently attached ferrocene was previously observed and described.^9,27–29^ An AzaPc core was selected due to its strong electron-deficient character (in comparison to parent Pcs), which is suitable as an acceptor for electron transfer processes.^7^

**Figure 1:**
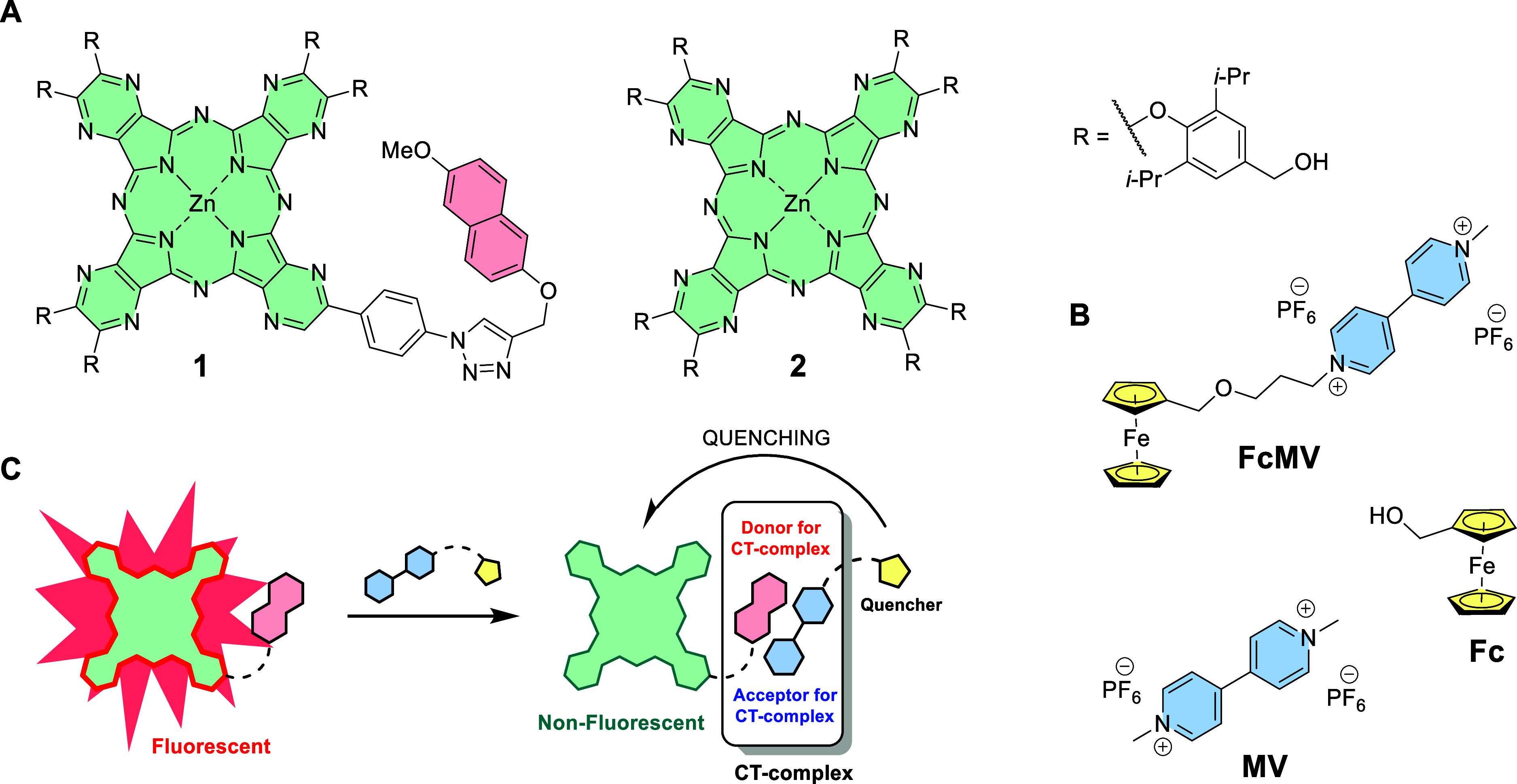
Structures of the studied (A) AzaPc derivatives **1** and **2** and (B) quenchers **FcMV**, **MV**, and **Fc**, and (C) a schematic representation of the principle of fluorescence quenching by a ferrocene-based quencher enhanced by formation of a CT-complex.

The structures of both AzaPc and the quencher were designed to contain moieties favoring their interaction by supramolecular forces in CT-complexes. The system involved an electron-deficient AzaPc core (fluorescent reporter, **1**) with the attached moiety derived from naphthalene-2,6-diol (donor in the CT-complex), and a quencher (**FcMV**) consisting of a quenching ferrocene moiety attached via a linker to a methylviologen derivative (acceptor in the CT-complex). The fluorescence quenching efficiency of **FcMV** was also compared to both individual components — methylviologen (in *N*,*N′*-dimethylviologen hexafluorophosphate, **MV**) and ferrocene (in ferrocenemethanol, **Fc**).

## Results and Discussion

### Synthesis

Synthesis of the final AzaPc derivative **1** started from preparation of its precursors **3** and **4** (Scheme 1). Compound **3** was prepared according to a previously published procedure from 5,6-dichloropyrazine-2,3-dicarbonitrile by its nucleophilic substitution by the corresponding phenol.^30^ Compound **4** was prepared from 4-aminoacetophenone in three steps. First, the amino group was converted into an azido group via diazonium salt, leading to 4-azidoacetophenone (**5**) in 94% yield. The next two steps were advantageously performed as a one-pot synthesis. The ketone was oxidized to the substituted glyoxal with SeO_2_ and then directly condensed with diaminomaleonitrile under acidic conditions to yield precursor **4** in 11% over these two steps.

**Scheme 1:**
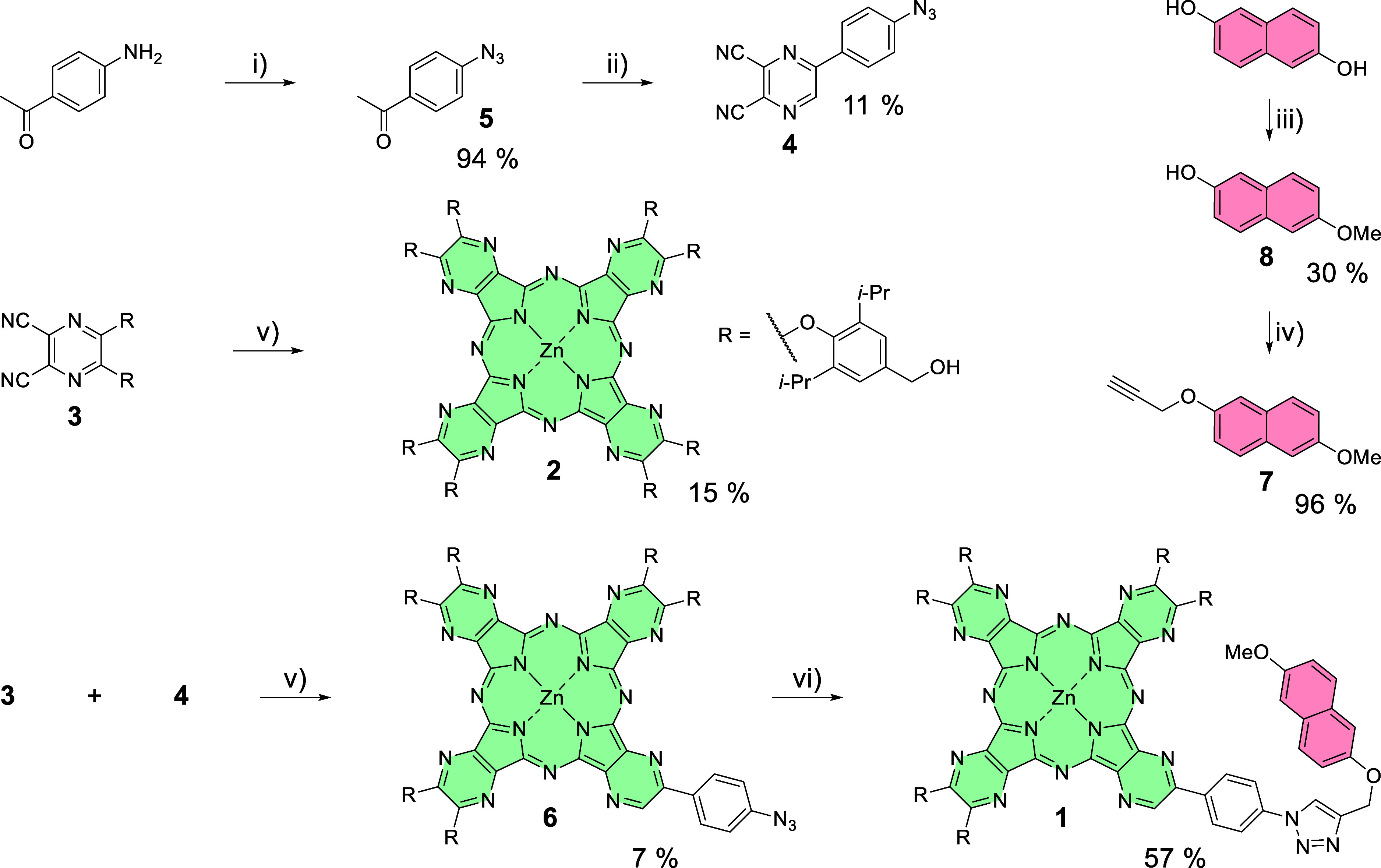
Synthesis of AzaPc Derivatives **1** and **2** and Their Precursors (**4**–**8**)­^a^

a (i) NaNO_2_, NaN_3_, HCl/H_2_O, 0 °C → rt; (ii) SeO_2_, 1,4-dioxane/H_2_O, reflux, then addition of conc. HCl and diaminomaleonitrile, reflux; (iii) K_2_CO_3_, MeI (1.3 equiv), acetone, reflux; (iv) K_2_CO_3_, propargyl bromide, MeCN, rt; (v) Zn­(OAc)_2_, pyridine, reflux; (vi) alkyne **7**, CuI, pyridine/DIPEA/MeCN/H_2_O, 40 °C.

Mixed cyclotetramerization (Scheme 1) of **3** (unit A) and **4** (unit B) in pyridine in the presence of zinc­(II) acetate provided a mixture of congeners from which the AAAB-type derivative (compound **6**) was isolated as the second intense green fraction by repeated column chromatography in 7% yield. The symmetrical AzaPc (AAAA type, compound **3**) was not isolated directly from this mixture because of the complicated purification of the obtained fractions. Rather, it was synthesized directly from precursor **3** under analogous conditions and obtained in 15% yield.

AzaPc **1** bearing a modified naphthalene-2,6-diol unit was synthesized using copper­(I)-catalyzed azide–alkyne cycloaddition (CuAAC) of AzaPc **6** with 2-propargyloxy-6-methoxynaphthalene (**7**). Derivative **7** was synthesized in two subsequent alkylations of naphthalene-2,6-diol. The first one with methyl iodide in slight excess gave 6-methoxynaphthalene-2-ol (**8**) in 30% yield. Then, **8** was alkylated with propargyl bromide in an almost quantitative yield. CuAAC of alkyne **7** and AzaPc **6** was performed with copper­(I) iodide in an emulsion of DIPEA, pyridine (for the solubility of AzaPc), MeCN, and water to get target AzaPc **1** in 57% yield. Its structure was confirmed mainly by HR MS giving the proper mass (Figure S14) as the signals in ^1^H NMR did not provide unequivocal confirmation of the structure (Figure S1). Moreover, IR spectroscopy confirmed that there was no band for the asymmetric vibration of the azido group in the spectrum of **1** in contrast to the spectrum of azido intermediate **6** (whose asymmetric vibration of the azido group was observed at 2122 cm^–1^, Figure S19). Additionally, HPLC analysis indicated the formation of the product eluted at a different retention time than the starting azide **6**. After purification by preparative TLC, AzaPc **1** reached over 94% purity according to HPLC (Figure S20), which was considered satisfactory for subsequent photophysical experiments.

For the synthesis of the quencher moiety, an alkylating reagent containing a ferrocene unit was prepared first. Thus, ferrocenemethanol (**Fc**) was modified by attaching a brominated linker, providing compound **9** in almost quantitative yield using chromatographically purified 3-bromopropan-1-ol in large excess as a solvent (Scheme 2).^31^ After that, a bromine atom was substituted for iodine, a better leaving group, in the Finkelstein reaction (92% of **10**). Quencher **FcMV** was subsequently prepared in two steps. First, *N*-methylviologen iodide (**11**) was prepared by methylation with 0.6 equiv of methyl iodide. The positively charged monomethylated product precipitated out from dichloromethane used as a solvent in 66% yield and did not participate in further alkylation to the dimethylated product. Compound **11** was alkylated with **10** in MeCN, and **FcMV** was obtained in 24% yield by precipitation with NH_4_PF_6_ in water. In contrast to **11**, the dimethylated analogue can be obtained under different reaction conditions using methyl iodide in excess (6 equiv) and MeCN as a more polar solvent. The final hexafluorophosphate salt (**MV**) was obtained in the same manner as for **FcMV** in 84%.

**Scheme 2:**
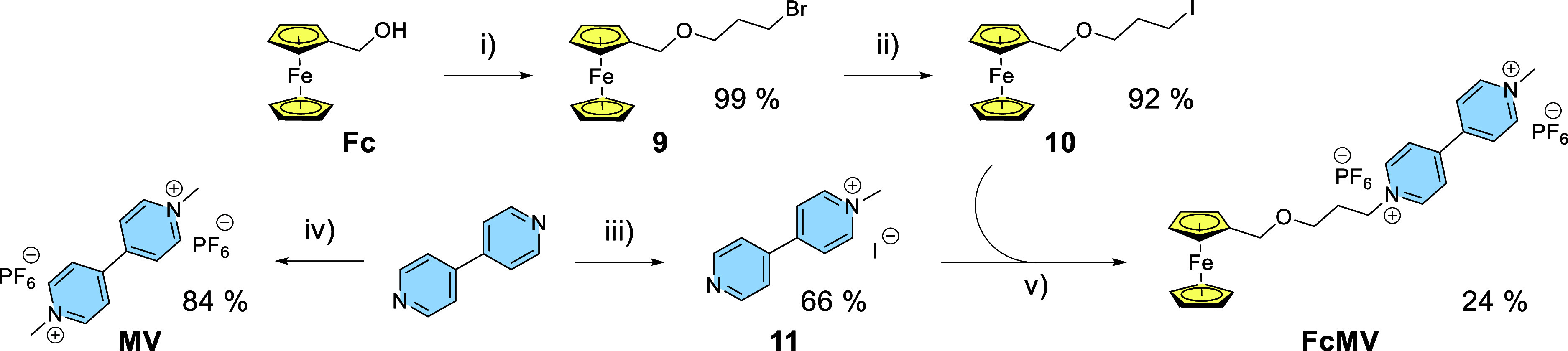
Synthesis of **FcMV**, Its Precursors (**9**–**11**), and **MV** ^a^

a (i) 3-Bromopropan-1-ol (excess), cat. AcOH, rt, (ii) NaI, acetone, reflux, (iii) MeI (0.6 equiv), DCM, rt, (iv) MeI (6 equiv), MeCN, reflux, then NH_4_PF_6_, H_2_O, (v) MeCN, reflux, then NH_4_PF_6_, H_2_O.

### Steady-State Fluorescence Measurements

AzaPc **1** has intense fluorescence in MeCN (Φ_F_ = 0.19, τ_F_ = 2.09 ns, λ_em_ = 642 nm) not differing from its azide precursor **6** (Φ_F_ = 0.22, τ_F_ = 2.61 ns, λ_em_ = 643 nm, Figure S24B) or symmetrical AzaPc **2** (Φ_F_ = 0.19, τ_F_ = 1.93 ns, λ_em_ = 638 nm, Figure S24A). This also confirms that the naphthalene-2,6-diol-derived moiety in **1** (a donor for the CT-complex) does not quench the excited state of AzaPc. On the basis of their absorption (Figure S23A), all three compounds were found in the monomeric form in MeCN without any observation of the aggregated species. This was further confirmed by the perfect accordance of the absorption and excitation spectra (Figures 2 and S24).

**Figure 2:**
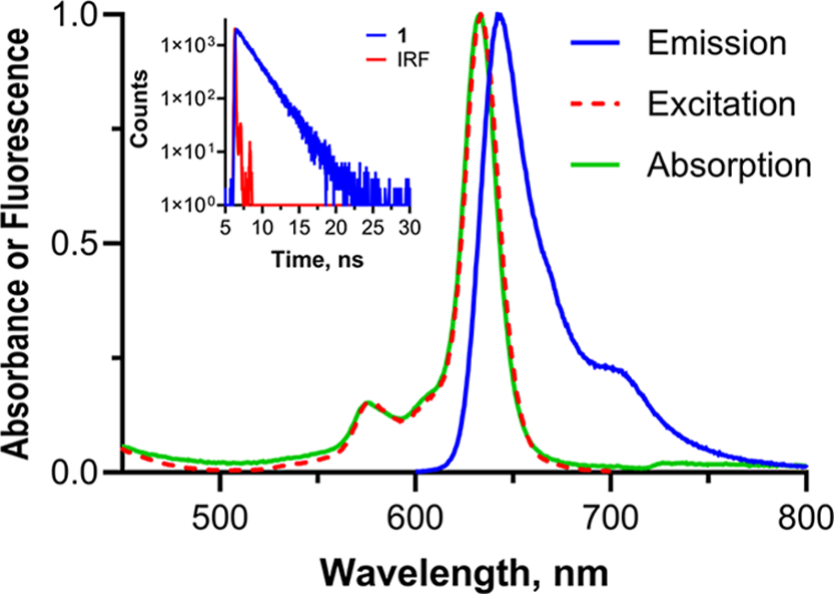
Normalized absorption (green line), excitation (dashed red line; emission was measured at 710 nm), and emission (blue line; excitation at 590 nm) spectra of **1** (1 μM, MeCN). The inset shows fluorescence decay during the lifetime measurement.

The benefit of the CT-complex formation for quenching of AzaPc fluorescence by ferrocene was subsequently tested in a series of experiments. First, the fluorescence emission of AzaPc **1** was monitored in MeCN upon addition of **FcMV** (the fluorescence changes were monitored at 646 nm) and compared to the similar titrations with controls **MV** and **Fc**. In all cases, addition of any of the quenchers led to a concentration-dependent decrease of fluorescence intensity (Figure 3A–C), which was, however, the most significant in the case of **FcMV**, indicating the strongest quenching. No changes in the shape of the absorption or emission spectra of **1** were observed even at the highest concentrations of the quenchers.

**Figure 3:**
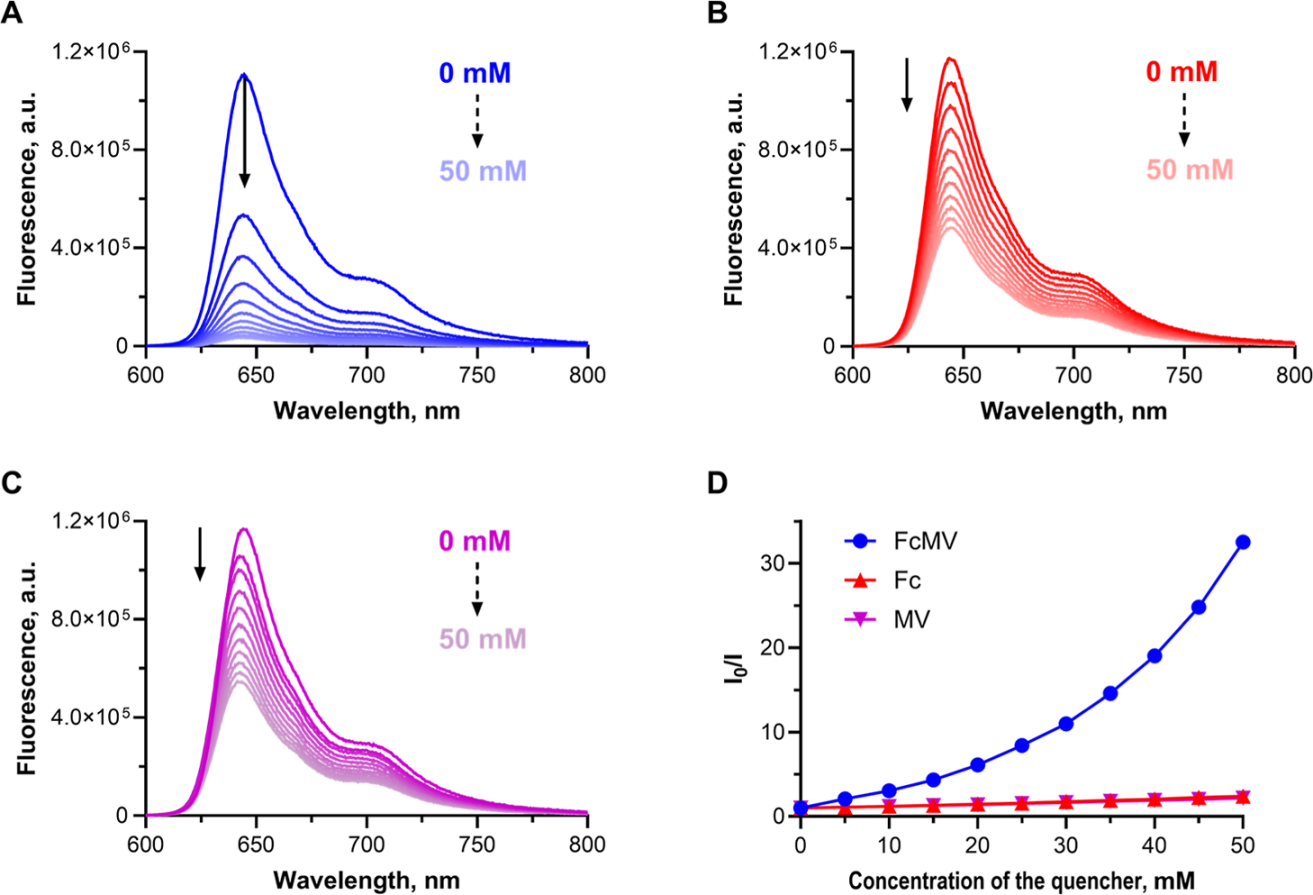
Fluorescence spectra of solution of **1** (5 μM, MeCN) with (A) **FcMV**, (B) **Fc**, and (C) **MV** of different concentrations in the range of 0–50 mM, and (D) Stern–Volmer plots of AzaPc quenching by **FcMV** (blue), **Fc** (red), and **MV** (purple) calculated from the intensities of fluorescence emission at 646 nm (*I*_0_ corresponds to the intensity of pure **1**; *I* is the intensity at the given concentration of the quencher; excitation wavelength was 590 nm).

The mechanism of quenching can be generally dynamic or static. Dynamic quenching is a diffusion-driven event as it happens upon collisions of the excited state with the quencher. Therefore, the fluorescence lifetime changes with the concentration of the quencher, while the shape of the absorption spectrum remains unchanged. Static quenching occurs when the quencher interacts with the emitter in the ground state to produce nonradiative species (e.g., a complex or protonated/deprotonated form). The number of nonquenched molecules available for fluorescence emission decreases, but their lifetime does not change. On the other hand, the shape of the absorption spectra is not a sum of the fluorophore and quencher but typically changes due to the formation of a new species.

The character of quenching can be evaluated by means of a Stern–Volmer plot correlating the ratio of the initial fluorescence intensity of the fluorophore and intensity in the solution with the quencher (*I*_0_/*I*) to the concentration of the quencher. In the Stern–Volmer plot, both pure static and pure dynamic quenching manifest in linear relationships, while a combination of both factors causes an upward deviation of this relationship.^32^

The data from the titrations of AzaPc **1** with **FcMV** were evaluated by means of a Stern–Volmer plot (emission at 646 nm, Figure 3D). The linear dependence was observed in experiments with **Fc** and **MV**, showing either pure static or pure dynamic quenching (Figure S26A; for further analysis, see below). However, the fluorescence of **1** in the solution of **FcMV** decreased more significantly, and the dependence of *I*_0_/*I* on the concentration of **FcMV** was clearly nonlinear. This suggests that both static (as a consequence of CT-complex formation and convergence of the ferrocene quencher moiety closer to AzaPc) and dynamic (collision) components participate in the interactions, leading to quenching. Formation of a CT-complex should potentially lead to changes in absorption spectra in the UV area; however, they were not detected due to the high concentration needed for CT formation. The changes in AzaPc absorption spectra were not observed, which was an expected result as the macrocycle is not conjugated to the binding moiety (naphthalene-2,6-diol moiety is connected by an aliphatic linker) and does not participate in the formation of the CT-complex.

### Time-Resolved Fluorescence Measurements

To distinguish between static and dynamic contributions to the quenching, the lifetimes of AzaPc **1** in the selected concentrations of quenchers’ solutions were measured (Table S1 and Figure S27). The lifetime of **1** was characterized by a monoexponential decay with τ_0_ = 2.09 ns. Upon the addition of **FcMV**, biexponential decay was observed. The shorter component did not change significantly with the concentration of the quencher, with τ_1_ = 0.2–0.4 ns, and can be attributed to static quenching by ferrocene after formation of the CT-complex. The longer lifetime (τ_2_) decreased with an increasing concentration of **FcMV** in the solution (down to τ_2_ = 1.00 ns at 50 mM concentration of **FcMV**). This second component was used for further calculations (τ_2_ = τ). The ratio τ_0_/τ depends linearly on the concentration of the quencher, which suggests a contribution from the dynamic character of the quenching. The slope of the linear relationship corresponds to the dynamic quenching constant (*K*_D_) and the product of bimolecular quenching rate constant (*k*_Q_) and τ_0_ according to the Stern–Volmer relationship (eq 1).^32^

1 $$\frac{\tau_{0}}{\tau }=1+K_{D}\left[Q\right]=1+k_{Q}\tau_{0}\left[Q\right]$$

The *K*_D_ value was calculated (Figure S28A) for quenching by **FcMV** (22.6 M^–1^) and **Fc** (18.9 M^–1^). The *k*_Q_ values were close to 1 × 10^10^ M^–1^ s^–1^ (for values, see Table 1), which suggests a diffusion-controlled process.^32^

**Table 1:** Bimolecular Excited Singlet State Quenching Rate Constants of the Dynamic Contribution (*k*_Q_), Dynamic (*K*_D_) and Static Quenching Constants (*K*_S_), and Association Constants (*K*_a_) of the Corresponding Complexes

	*k*_Q_ [M^–1^ s^–1^]	*K*_D_ [M^–1^]	*K*_S_ [M^–1^]	*K*_a_ [M^–1^]
**FcMV**	1.08 × 10^10^	22.6	241	174
**Fc**	9.04 × 10^9^	18.9		13.5
**MV**			22.5	14.5

A modified Stern–Volmer relationship (eq 2) containing both dynamic quenching constant (*K*_D_) and static quenching constant (*K*_S_) can be used to describe situations in which both static and dynamic quenching occur.^32^ Knowing the *K*_D_ from the time-resolved fluorescence measurements, it is possible to fully describe the nonlinear relationship observed in the steady-state Stern–Volmer plot (Figure 3D).

2 $$\frac{I_{0}}{I}=1+\left(K_{D}+K_{S}\right)\left[Q\right]+\left(K_{D}K_{S}\right){\left[Q\right]}^{2}$$

The nonlinear regression (Figure S28C) showed that the quenching of **1** by **FcMV** can be described by a *K*_S_ of 241 M^–1^. Since lifetime values did not change in the experiments with **MV**, the observed quenching has a static character. For this system, the Stern–Volmer relationship (eq 3) can be used. The value was calculated (Figure S28B) directly from the Stern–Volmer plot (Figure 3D, 22.5 M^–1^).

3 $$\frac{I_{0}}{I}=1+K_{S}\left[Q\right]$$

The data presented in Table 1 and Stern–Volmer plots (Figure 3D) show that quenching by **FcMV** was improved compared to quenching by **Fc** itself. The dynamic quenching by both compounds was comparable; however, the static component in the case of **FcMV** improved the quenching dramatically, indicating strong support of the CT-transfer complex formation that brought the **Fc** unit into the proximity of the AzaPc core. Moreover, it was shown that the contribution to the quenching of the methylviologen moiety itself is negligible (as expected) because **MV**, lacking the ferrocene unit, did not quench **1** efficiently.

The values of quenching constants can also be compared to the association constants of the complexes of the AzaPc derivative and the quencher because both processes, binding of the quencher by the naphthalene-2,6-diol moiety and the quenching of the AzaPc fluorescence by the static quenching contribution, are related. The association constant (*K*_a_, eq 4) is an equilibrium constant of the formation of a complex between AzaPc and a quencher (AzaPc + Q ⇌ AzaPc·Q). If the concentration of the quencher is much higher than the concentration of AzaPc as in our case, the equilibrium concentration of the complex [AzaPc·Q] is negligible compared to the equilibrium concentration of the free quencher [Q]. Therefore, [Q] is approximately equal to analytical concentration (*c*_Q_ = [Q] + [AzaPc·Q]≈[Q]).

4 $$K_{a}=\frac{\left[AzaPc\cdot Q\right]}{\left[AzaPc\right]\left[Q\right]}\approx \frac{\left[AzaPc\cdot Q\right]}{\left[AzaPc\right]c_{Q}}$$

The observed intensity of the fluorescence (at 646 nm) at any time of the titration (*I*_obs_) is equal to the sum of the intensity of the noncomplexed form of AzaPc (*I*_AzaPc_) and the complexed form (*I*_AzaPc·Q_) according to eq 5, where *c*_AzaPc_ is the analytical concentration of AzaPc and the sum of equilibrium concentrations of AzaPc and quencher (*c*_AzaPc_ = [AzaPc] + [AzaPc·Q]).

5 $$I_{obs}=I_{AzaPc}\frac{\left[AzaPc\right]}{c_{AzaPc}}+I_{AzaPc\cdot Q}\frac{\left[AzaPc\cdot Q\right]}{c_{AzaPc}}$$

By combining eqs 4 and 5, the association constant can be calculated from eq 6,^33^ where Δ*I* is the difference in the fluorescence intensity (*I*) at any point of the titration from the initial intensity (*I*_0_) at zero concentration of the quencher, and Δ*I* ^∞^ is the difference of extrapolated intensity at complete complexation from *I*_0_.

6 $$\Delta I=I-I_{0}=\Delta I^{\infty }\frac{K_{a}c_{Q}}{1+K_{a}c_{Q}}$$

The resulting value of *K*_a_ = 174 M^–1^ (Table 1 and Figure S28D) for **FcMV** roughly corresponds to the value of *K*_s_ = 241 M^–1^ for **FcMV**.

Further experiments were conducted with symmetrical AzaPc **2** (Figures 4 and S25 for fluorescence emission spectra) to verify the impact of the naphthalene-2,6-diol moiety and to evaluate whether other parts of the molecule (4-hydroxymethyl-2,6-diisopropylphenoxy substituents) may also contribute to binding. As expected, the decrease in the fluorescence of **2** in solutions of controls **Fc** and **MV** was similarly low to the values of **1**. In contrast, the behavior of both AzaPcs in the presence of **FcMV** differed, with AzaPc **1** being quenched significantly more efficiently than AzaPc **2**. This suggests that 4-hydroxymethyl-2,6-diisopropylphenoxy substituents may also be responsible for the formation of the CT-complex with **FcMV**. However, the difference between these two AzaPcs emphasizes the impact of the naphthalene-2,6-diol group, enabling the formation of stronger CT-complexes. To further support this conclusion, the association constant of **2** with **FcMV** was calculated in the same manner as for **1** from the fluorescence intensities (Figure S28D). The value of *K*_a_(**2**) = 101 M^–1^ is lower than that of *K*_a_(**1**) = 174 M^–1^, showing that the complex with the viologen part of the quencher is less thermodynamically favored.

**Figure 4:**
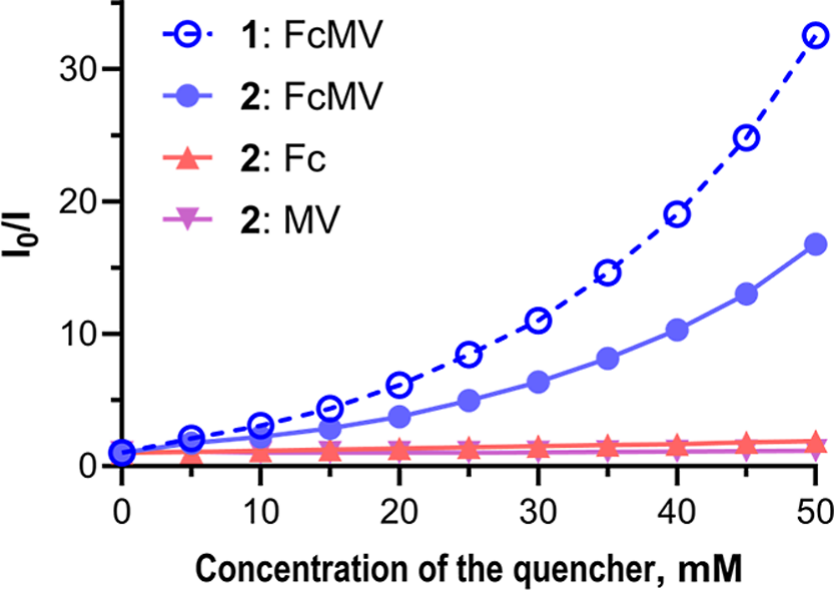
Stern–Volmer plots calculated from the intensities of fluorescence emission at 646 nm (*I*_0_ corresponds to the intensity of pure **1**; *I* is the intensity at the given concentration of the quencher; excitation wavelength was 590 nm) from experiments testing the fluorescence of symmetrical AzaPc **2** (5 μM, MeCN) in solution with **FcMV** (blue), **Fc** (red), and **MV** (purple) of different concentrations in the range of 0–50 mM in comparison to AzaPc **1** (5 μM, MeCN) in the solutions with **FcMV** (blue, dashed line).

### Electrochemistry

Several literature reports suggest that the quenching mechanism of ferrocene-substituted Pcs is based on photoinduced electron transfer (PET).^27,29^ Since AzaPcs are known to have even more electron-deficient character compared to Pcs and that ferrocene can be simply oxidized (*E*_ox_ = 0.38–0.56 V vs SCE, depending on the solvent),^34^ a similar mechanism can be expected in our system as well once the moieties are snapped together by CT-complex staple (in static quenching). To confirm that, the analysis of the thermodynamic feasibility of the electron transfer process by means of electrochemistry was performed. The cyclic voltammetry (CV) and square-wave voltammetry (SWV) were measured in MeCN to reflect the conditions identical to the above-mentioned fluorescence titration experiments. However, the solubility of AzaPc **1** in MeCN was found to be insufficient to reach concentrations required for electrochemistry, and consequently, reduction and oxidation waves in CV were not well resolved, barely distinguishable from the blank. Therefore, the solvent was changed to pyridine, enabling an unequivocal assignment of the redox potentials (Table 2).

**Table 2:** Half-Wave Redox Potential Data for AzaPc **1** and **FcMV** ^a^

	*E*_ox_^1^, V vs SCE	*E*_ox_^2^, V vs SCE	*E*_red_^1^, V vs SCE	*E*_red_^2^, V vs SCE
AzaPc **1**	0.61	0.97	–0.66	–0.98
**FcMV**	0.57		–0.29	–0.75

a Potentials *E*_red_ and *E*_ox_ were measured in pyridine and obtained from square-wave voltammograms. They are expressed as *E*_1/2_ (in V vs SCE) with Fc/Fc^+^ as an internal standard.

The cyclic voltammograms of **FcMV** (see Figure 5B) show the same electrochemical properties as those of individual ferrocene or methylviologen (Figure S31). It possesses a reversible oxidation with the oxidation potential (*E*_ox_) at 0.57 V vs SCE corresponding to the oxidation of the ferrocene moiety and two reversible reductions with reduction potentials (*E*_red_) at −0.29 and −0.75 V vs SCE corresponding to the methylviologen part. This also proved that both moieties in **FcMV** are electrochemically independent. AzaPc **1** exerted two oxidation and two reduction potentials within the potential window of the solvent. The first reduction potential (*E*_red_^1^) at −0.66 V vs SCE indicates a significantly electron-deficient character of the AzaPc core in comparison with Pcs, where the first reduction potentials typically lie above 1.0 V vs SCE.^7,34^

**Figure 5:**
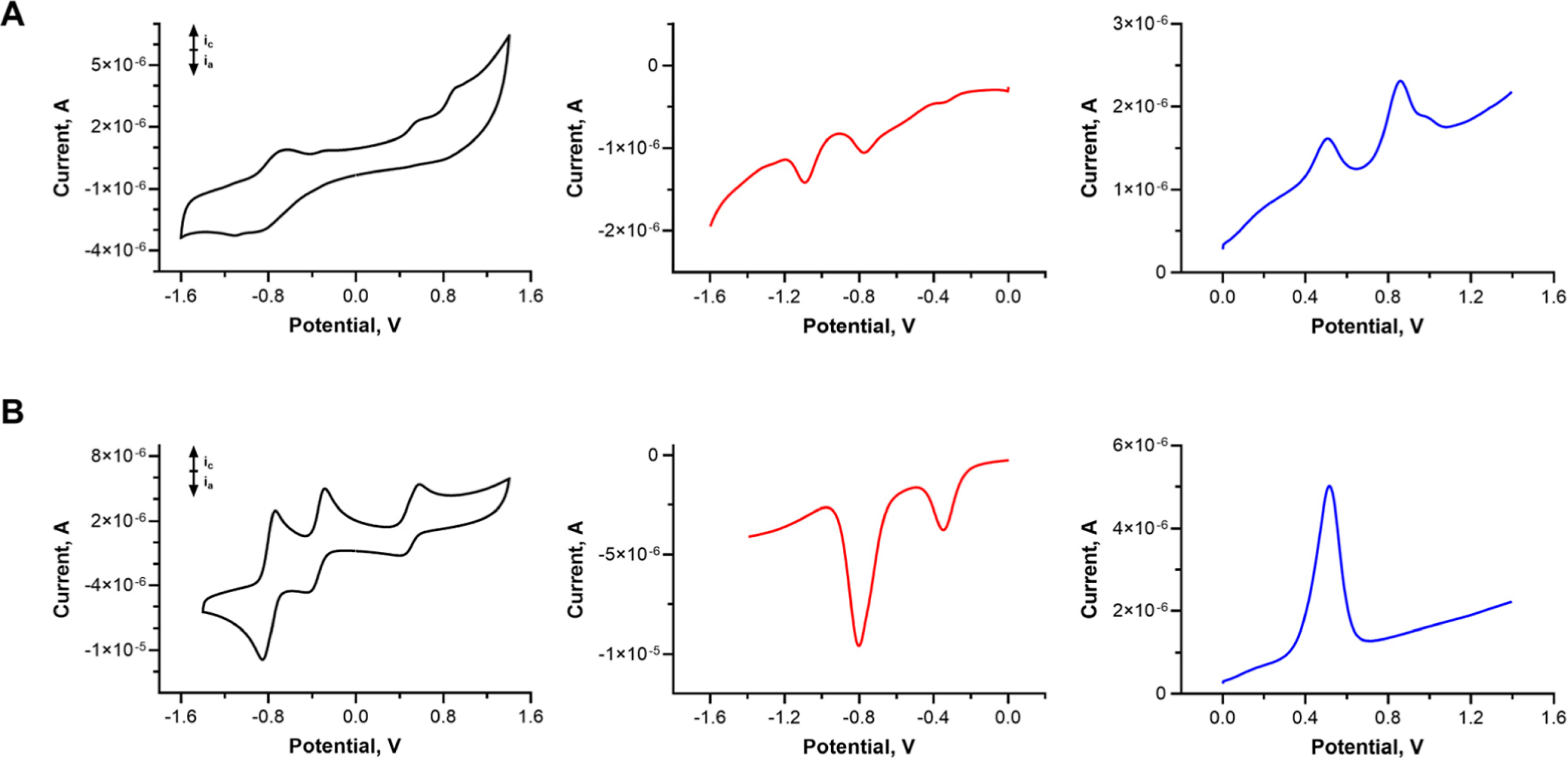
Cyclic voltammograms (left, black) and square-wave voltammograms (reduction-middle, red; oxidation-right, blue) in pyridine (100 mV/s, 0.1 M tetrabutylammonium hexafluorophosphate as the supporting electrolyte, 25 °C) of AzaPc **1** (A) and **FcMV** (B). Potential vs SCE was determined according to the oxidation of ferrocene used as an internal standard, *E*(Fc/Fc^+^) = 0.56 V vs SCE;^34^ solutions of the presented voltammograms do not contain ferrocene as an internal standard.

The obtained electrochemical data were used subsequently for estimation of the thermodynamic feasibility of the electron transfer process (the most probable quenching mechanism of the static quenching in our system) by determination of the Gibbs energy change of PET (eq 7)^32^

7 $$\Delta G^{{}^{\circ}}=E_{1/2}\left(D^{+}/D\right)-E_{1/2}\left(A/A^{-}\right)]-E_{00}-w_{p}$$

where Δ*G* ^0^ is the free energy change for electron transfer. *E*_1/2_(D^+^/D) and *E*_1/2_(A/A^–^) are the half-wave reduction potentials of the donor (D^+^/D) and acceptor (A/A^–^) couples (taken from Table 2), respectively, and *E*_00_ is the energy required for photoexcitation from S_0_ to S_1_ (determined to be 1.93 eV for AzaPc **1** in pyridine from the average values of the *Q*_*x*_(0,0) absorption and *Q*(0,0) emission bands). The *w*_p_ is a Coulombic interaction between the oxidized donor and the reduced acceptor, which is typically small and can be neglected in solvents with a high dielectric constant (e.g., in pyridine).^27^ Based on the calculated data, strongly negative Δ*G* ^0^ = – 0.70 eV was obtained. It indicated that the electron transfer process is thermodynamically favorable. The value is even more negative than the free energy changes published for Pc^27^ or porphyrins^35,36^ with a covalently attached ferrocene moiety which is a consequence of the strongly electron-deficient AzaPc core.

### Transient Absorption Spectroscopy

After the thermodynamic feasibility of the electron transfer process was confirmed, we attempted to observe the potentially involved species by transient absorption spectroscopy (TAS). The excited singlet state of AzaPc **1** or **2** was observed upon 640 nm laser excitation (Figures 6 and S32). The measured data were globally analyzed with a sequential model, and the excited singlet state showed triexponential decay after analysis, probably caused by the inhomogeneity of the sample (due to possible aggregation, unequal distribution of the dye in the sample, and high concentration of the sample used compared to the emission measurements). The observed lifetimes of the excited singlet state for both dyes were in units, hundreds of picoseconds and 2 ns, respectively, with the longest component corresponding to the data obtained from time-resolved fluorescence experiments. The first two ps lifetime components primarily reflect the static self-quenching of the singlet excited state of AzaPc **1** or **2** (Figures 6A,B and S32A,B). This self-quenching, estimated by comparing the intensity of the ground-state bleach at 630 nm, corresponds to approximately 25% of the excited molecules being self-quenched. The rest of the singlet excited state molecules underwent intersystem crossing to the triplet state (last spectra in Figure 6) whose lifetime was longer than the measurement window of the picosecond TAS (hundreds of nanoseconds). Upon addition of the **FcMV** quencher (*c* = 12.5 or 50 mM, Figures 6C,D and S32C,D), no new absorption appeared within the measurement window, indicating that no new species (e.g., AzaPc anion radical) were detected, possibly due to the low quantum yield of the electron transfer. The only effect observed was a shortening of all three lifetimes of the S_1_ state. The picosecond lifetimes of S_1_ were shortened to 4 and 40 ps via static quenching, corresponding to more than 50% of excited molecules being quenched. The longest S_1_ lifetime component was dynamically quenched to 1 ns or 560 ps at AzaPc **1** concentrations of 15 and 30 mM, respectively, which is also in line with the above-referred time-resolved fluorescence experiments. Therefore, although TAS measurements were in accordance with time-resolved emission experiments, they could not unequivocally confirm (or rule out) the presence of new species and the PET mechanism as the main mechanism of the static quenching. Of note, the addition of **FcMV** at high concentrations leads to precipitation of AzaPc **1** (at both tested concentrations) from the solution after a short period of time, which might also affect the measurement by decreasing the homogeneity of the system.

**Figure 6:**
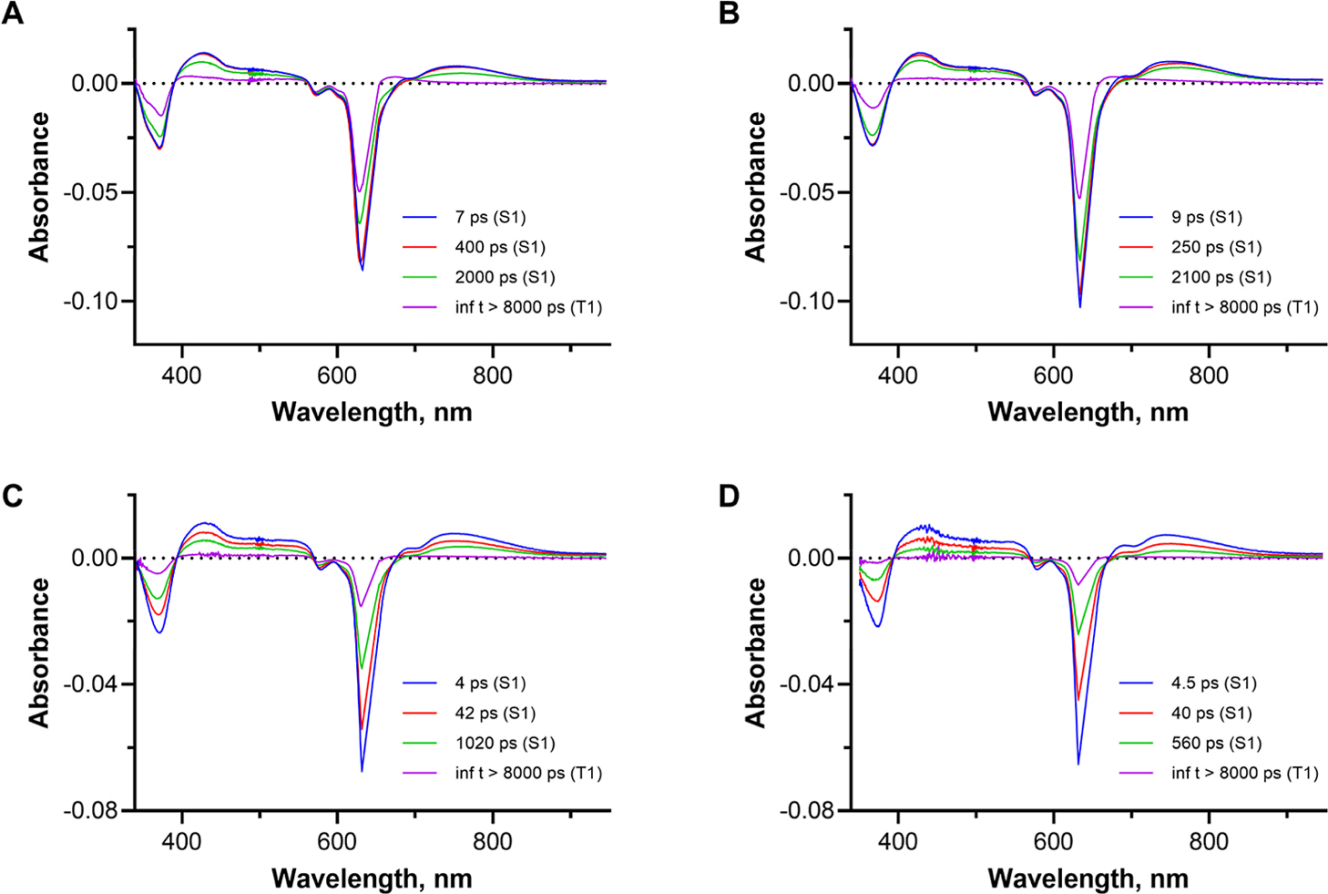
Species-associated spectra with corresponding lifetimes obtained from global analysis of TAS data in MeCN (nondegassed). (A) AzaPc **2**, (B) AzaPc **1**, (C) AzaPc **1** (15 μM) + **FcMV** (12.5 mM), and (D) AzaPc **1** (30 μM) + **FcMV** (50 mM), sequential model used. Samples were excited at 640 nm and 200 nJ pump energy. The first three species are the singlet excited state of the dye, and the fourth species is the triplet excited state of the dye.

## Conclusions

In summary, a peripherally substituted AzaPc and a ferrocene-based quencher were designed to interact through noncovalent CT-complex formation, offering a new approach to enhance quenching efficiency. The system was described by means of static and dynamic quenching constants, with the former being significantly higher. The static quenching constant roughly corresponds to the association constant of the charge-transfer complex (2 × 10^2^ M^–1^) of AzaPc **1** and the quencher **FcMV**. Fluorescence measurements of AzaPc **1** and symmetrical AzaPc **2** demonstrated that the incorporation of a naphthalene-2,6-diol moiety significantly enhances AzaPc’s affinity for quenchers modified by a viologen motif. Notably, the quenching efficiency of the ferrocene-based quencher was markedly improved through conjugation with methylviologen, enabling the formation of noncovalent CT-complexes. The exact mechanism of static quenching was not directly confirmed, although PET from the ferrocene donor to the AzaPc acceptor seems to be a plausible and thermodynamically feasible explanation. These findings provide valuable insights into the rational design of supramolecular donor–acceptor systems and offer a promising strategy for fine-tuning photoactive materials, although still with a relatively low association constant in the presented system in organic solvents. The fact that the CT-complexes can indeed increase the quenching efficiency opens possibilities to use these interactions in the design of advanced derivatives, e.g., in ternary complexes with cucurbit[8]­uril in aqueous media, while reaching potentially significantly higher association constants.

## Experimental Part

### General Information

Chemicals were purchased from commercial suppliers (Sigma-Aldrich, Fluorochem, BLDpharm, Lach-Ner, Strem) and used without further purification or treatment except for ferrocenemethanol, 3-bromopropan-1-ol, and K_2_CO_3_. Ferrocenemethanol for titrations was dissolved in MeCN, filtered, and evaporated on a rotary evaporator. 3-Bromopropan-1-ol was purified by column chromatography with hexane/EtOAc as the mobile phase, and the TLCs were visualized with KMnO_4_ stain (1.5 g of KMnO_4_, 10 g of K_2_CO_3_, and 1.25 mL of 10% NaOH in 200 mL of water). K_2_CO_3_ for alkylations was dried in an oven overnight at 120 °C. Organic solvents used for the synthesis were of analytical grade. MeCN for photophysical studies and HPLC analysis was LC–MS grade. TLCs were run on Merck aluminum sheets coated with Silica gel 60 F254. Chromatography columns were packed with Merck Kieselgel 60 (0.040–0.063 mm). Compounds **3**,^30^ **8**,^37^ and **11** ^38^ were synthesized according to published procedures.

The NMR spectra were recorded on a Varian VNMR S500 NMR spectrometer (Agilent Technologies, Santa Clara, USA) or Jeol JNM-ECZ600R (Jeol, Akishima, Japan). The samples of low-molecular-weight compounds were dissolved in CDCl_3_, CD_3_CN, or DMSO-*d*_6_. The samples of AzaPcs were measured in mixture of CDCl_3_ and pyridine-*d*_5_ (3:1, v/v) to prevent aggregation based on the previous experience with a similar compound to compare with literature data (AzaPc **2**). The chemical shifts (δ) are given in parts per million (ppm); coupling constants (*J*) are given in Hertz (Hz). The multiplicities are reported as singlet (s), doublet (d), triplet (t), quartet (q), doublet of doublets (dd), or multiplet (m). The ^1^H and ^13^C spectra are referenced to the solvent residual peak, and the signals are assigned to corresponding nuclei using 2D experiments (HSQC, HMBC). The IR spectra were measured on a Nicolet 6700 (Thermo Scientific, USA). The wavenumbers (ν̃) are given in reciprocal centimeters (cm^–1^). The UV/vis spectra were recorded on a Shimadzu UV-2600 spectrophotometer (Shimadzu, Kyoto, Japan). The fluorescence spectra were recorded on an FLS-1000 Photoluminescence Spectrometer (Edinburgh Instruments, Edinburgh, United Kingdom). Wavelengths (λ) are given in nanometers (nm), and extinction coefficients (ε) are in dm^3^ mol^–1^ cm^–1^. High-resolution mass spectra (HRMS) were measured on a UHPLC system Acquity UPLC I-class (Waters, Millford, USA) coupled to an HRMS Synapt G2Si (Waters, Manchester, UK) based on Q-TOF. Chromatography for HRMS was carried out using an Acquity UPLC Protein BEH C4 (2.1 × 50 mm, 1.7 μm, 300 Å) column using gradient elution with MeCN and 0.1% formic acid at a flow rate of 0.4 mL/min. Electrospray ionization was operated in the positive or negative ion mode. The ESI spectra were recorded in the range of 50–5000 *m*/*z* using leucine–enkephalin as a lock mass reference and sodium iodide for external calibration or in the range of 50–1200 *m*/*z* using leucine–enkephalin as a lock mass reference and sodium formate for S6 external calibration.

### Synthesis

#### AzaPc **1**

AzaPc **6** (27.5 mg, 0.0142 mmol, 1.0 equiv), 2-methoxy-6-propargyloxy-naphthalene (30.6 mg, 0.144 mmol, 10 equiv), and CuI (4.2 mg, 0.022 mmol, 1.6 equiv) were dissolved in pyridine (3 mL). DIPEA (3 mL) and MeCN (3 mL) were added, followed by the addition of water (3 mL). The emulsion was stirred at 40 °C for ca. 18 h. The reaction mixture was carefully evaporated on a rotary evaporator and purified by column chromatography using a DCM/MeOH/pyridine (290:10:3, v/v/v) mixture as the mobile phase. The column was first washed with pure DCM to recover the excess of 2-methoxy-6-propargyloxy-naphthalene, then the green fraction was eluted with the mobile phase mentioned above (for TLC, seeFigure S30). The final product dissolved in pyridine (0.350 mL) was further purified by precipitation with hexane (7 mL). The precipitate was separated by centrifugation and washed with hexane (1 × 7 mL) and Et_2_O (1 × mL). The residual solvents were evaporated on a rotary evaporator at 60 °C. Yield: 57% (17.5 mg) of a dark green solid. ^1^H NMR (600 MHz, CDCl_3_ and pyridine-*d*_5_ (3:1, v/v), 25 °C): δ 9.80 (s, 1H), 8.74 (d, *J* = 8.1 Hz, 2H), 8.28 (d, *J* = 8.0 Hz, 2H), 7.64 (d, *J* = 2.0 Hz, 12H), 7.64–7.63 (m, 4H), 5.26 (s, 8H), 5.11–5.04 (m, 4H), 3.61–3.45 (m, 12H), 1.56–1.32 (m, 72H). ^13^C NMR (151 MHz, CDCl_3_ and pyridine-*d*_5_ (3:1, v/v), 25 °C) δ 152.01, 151.70, 151.62, 151.52, 150.82, 150.73, 150.04, 149.60, 146.76, 146.65, 142.77, 142.48, 140.77, 140.68, 140.54, 140.21, 129.14, 120.63, 64.69, 64.54, 28.17, 23.35. IR-ATR ν̃ 3355, 2968 (Ar CH), 2374, 2309, 1660, 1565, 1398, 1245, 1102, 1051, 928, 689, 663, 614 cm^–1^. HRMS (ESI+): *m*/*z* calcd for [C_122_H_131_N_19_O_14_Zn + H^+^]: 2150.9487, found: 2150.9473. UV/vis (MeCN) λ (ε) 633 (131,903), 576 (19,047), 372 (82,945).

*AzaPc* **2** was synthesized following the same procedure as for AzaPc **6** using just precursor **3** instead of the mixture of **3** and **4** (precursor **3**: 185 mg, 0.341 mmol, 1.0 equiv, Zn­(OAc)_2_: 67.6 mg, 0.368 mmol, 1.1 equiv; yield: 29.2 mg, 15%). ^1^H NMR (600 MHz, CDCl_3_ and pyridine-*d*_5_ (3:1, v/v), 25 °C): δ 7.57 (s, 16H), 5.21 (s, 16H), 3.43 (p, *J* = 6.4 Hz, 16H), 1.39–1.31 (m, 96H).^13^C NMR (151 MHz, CDCl_3_ and pyridine-*d*_5_ (3:1, v/v), 25 °C): δ 151.42, 149.89, 146.63, 142.46, 140.61, 140.03, 64.53, 28.08, 23.25. UV/vis (MeCN) λ (ε) 627 (84,610), 572 (13,053), 371 (58,510). The NMR spectroscopic data correspond to the literature.^30^

#### 5-(4-Azidophenyl)­pyrazine-2,3-dicarbonitrile (**4**)

Compound **5** (4.93 g, 30.6 mmol, 1.0 equiv) and SeO_2_ (6.86 g, 61.8 mmol, 2.0 equiv) were dissolved in 1,4-dioxane (45 mL) and water (5 mL). The reaction mixture was refluxed (temperature of the heating block = 105 °C) for ca. 18 h. It was allowed to cool to rt, and diaminomaleonitrile (6.64 g, 61.4 mmol, 2.0 equiv) and conc. hydrochloric acid (6.1 mL) were added. The reaction mixture was heated again and refluxed (temperature of the heating block = 105 °C) for an additional 2 h. It was allowed to cool to rt, filtered, and evaporated on a rotary evaporator. The residue was suspended in acetone (100 mL) and collected by filtration; another batch was obtained from the filtrate after some acetone evaporated spontaneously. This process was repeated until 5 batches were collected. According to quantitative NMR, batches 2–5 (pale brown powder) reached purity over 90%; however, batch 1 (dark brown powder) contained around 50% of the desired product. Yield (batches 2–5): 11% (0.867 g). ^1^H NMR (500 MHz, DMSO-*d*_6_, 25 °C): δ 9.71 (s, 1H), 8.31 (d, *J* = 8.7 Hz, 1H), 7.35 (d, *J* = 8.7 Hz, 1H). ^13^C NMR (126 MHz, DMSO-*d*_6_, 25 °C) δ 152.55, 144.88, 143.90, 133.02, 130.64, 129.83, 129.34, 120.25, 114.48, 114.15. IR-ATR ν̃ 2406, 2241 (CN), 2121 (N_3_), 2090 (N_3_), 1600, 1548, 1437, 1326, 1308, 1290, 1205, 1128, 861, 669 cm^–1^. HRMS was not possible to measure due to low ionization or the observed instability of the compound in solution. m.p.: decomposition (color change: ca. 160 to 167 °C, brown to dark gray-brown, ca. 167 to 180 °C dark gray-brown powder to black liquid).

#### 4-Azidoacetophenone (**5**)

4-Aminoacetophenone (9.02 g, 66.8 mmol, 1.00 equiv) was suspended in water (30 mL) and conc. hydrochloric acid (30 mL). The suspension was cooled to 0–4 °C in an ice bath. NaNO_2_ (4.87 g, 70.6 mmol, 1.06 equiv) in water (22 mL) was added dropwise, followed by dropwise addition of NaN_3_ (4.56 g, 70.2 mmol, 1.05 equiv.; Danger: NaN_3_ belongs to the category Acute Tox. One for dermal exposition and Acute Tox. Two for inhalation and oral exposition, use appropriate personal protective equipment and take necessary precautions, contact of NaN_3_ with acids must be avoided as it may generate an increased explosion risk) in water (1 mL). The reaction mixture was stirred at rt for about 18 h. The reaction mixture was diluted with 100 mL of water and extracted to Et_2_O (150 mL + 2 × 100 mL). Combined organic layers were washed with water (150 mL) and brine (150 mL), dried over anhydrous Na_2_SO_4_, filtered, and evaporated. The product was purified by column chromatography on the normal phase using a hexane/EtOAc (4:1, v/v) mixture as the mobile phase. Yield: 94% (10.16 g) of a yellowish dense liquid that solidified. ^1^H NMR (500 MHz, CDCl_3_, 25 °C): δ 7.96 (d, *J* = 8.8 Hz, 2H), 7.08 (d, *J* = 8.9 Hz, 2H), 2.58 (s, 3H). ^13^C NMR (126 MHz, CDCl_3_, 25 °C) δ 196.67, 145.03, 133.96, 130.39, 119.09, 26.60. The spectroscopic data correspond to the literature.^39^

#### AzaPc **6**

A flask with **3** (1.43 g, 2.63 mmol, 3.0 equiv), **4** (0.215 g, 0.868 mmol, 1.0 equiv), and zinc acetate (0.639 g, 3.48 mmol, 4.0 equiv) was evacuated and flushed with argon. Anhydrous pyridine (5.0 mL) was added with a syringe, and the reaction mixture was refluxed (temperature of the heating block = 120 °C) and protected from light for 6 h under an inert atmosphere. Pyridine was evaporated on a rotary evaporator; the residue was suspended in 80 mL of water, and a dark green precipitate was collected by filtration and washed with water (5 × 10 mL). The monofunctionalized congener of AAAB type was separated by repeated column chromatography using a DCM/MeOH/pyridine (195:5:2, v/v/v) mixture as the mobile phase as the second intense green fraction (see Figure S29 for TLC). Yield: 7.2% (0.122 g) of a dark green solid. ^1^H NMR (600 MHz, CDCl_3_ and pyridine-*d*_5_ (3:1, v/v), 25 °C): δ 9.73 (s, 1H), 8.55 (d, *J* = 8.5 Hz, 2H), 7.64–7.60 (m, 12H), 7.41 (d, *J* = 8.3 Hz, 2H), 5.27–5.23 (m, 8H), 5.08–5.03 (m, 4H), 3.60–3.40 (m, 12H), 1.49–1.34 (m, 72H). ^13^C NMR (151 MHz, CDCl_3_ and pyridine-*d*_5_ (3:1, v/v), 25 °C): δ 152.20, 152.11, 151.83, 150.79, 150.76, 150.72, 150.67, 150.62, 150.38, 150.23, 149.80, 148.09, 147.12, 146.97, 146.90, 142.94, 142.89, 142.83, 142.50, 141.01, 140.89, 140.80, 140.38, 140.35, 133.20, 119.94, 77.99, 77.77, 77.56, 64.90, 64.79, 29.75, 28.41, 28.38, 23.54. IR-ATR ν̃ 3315, 2965 (Ar CH), 2122 (N_3_), 2092 (N_3_), 1602, 1541, 1401, 1245, 1100, 928, 749, 683, 636, 612 cm^–1^. HRMS (ESI+): *m*/*z* calcd for [C_108_H_119_N_19_O_12_Zn + H^+^]: 1938.8650, found: 1938.8621. UV/vis (MeCN) λ (ε) 632 (107,243), 576 (15,067), 372 (67,747).

#### 2-Methoxy-6-propargyloxynaphthalene (**7**)

A flask with **8** (0.162 g, 0.930 mmol, 1.0 equiv) and K_2_CO_3_ (0.303 g, 2.19 mmol, 2.4 equiv) was evacuated and flushed with argon. Anhydrous MeCN (2 mL) and propargyl bromide (89%, 0.13 mL, 1.4 mmol, 1.5 equiv) were added with a syringe. The reaction mixture was stirred at rt for approximately 18 h under an inert atmosphere. Water (10 mL) was added, and the reaction mixture was extracted with EtOAc (3 × 10 mL). The combined organic layers were washed with water (2 × 10 mL) and brine (10 mL), dried over anhydrous Na_2_SO_4_, filtered, and evaporated. The product was dissolved in a small amount of DCM (2 mL) and filtered through a silica gel pad. The product was eluted with DCM (3 × 30 mL), and fractions with the product were collected (TLC, mobile phase: DCM). Yield: 96% (0.139 g) of a white solid. ^1^H NMR (600 MHz, DMSO-*d*_6_, 25 °C): δ 7.75 (d, *J* = 9.2 Hz, 1H), 7.72 (d, *J* = 8.9 Hz, 1H), 7.33 (d, *J* = 2.7 Hz, 1H), 7.28 (d, *J* = 2.6 Hz, 1H), 7.16 (dd, *J* = 8.9, 2.6 Hz, 1H), 7.14 (dd, *J* = 8.9, 2.6 Hz, 1H), 4.87 (d, *J* = 2.4 Hz, 2H), 3.84 (s, 3H), 3.58 (t, *J* = 2.4 Hz, 1H). ^13^C NMR (151 MHz, DMSO-*d*_6_, 25 °C) δ 155.88, 153.50, 129.75, 129.09, 128.19, 118.87, 118.70, 107.95, 106.13, 79.35, 78.20, 55.49, 55.10. IR-ATR ν̃ 3280, 2964, 2934, 2856, 3131, 1603, 1508, 1395, 1373, 1229, 1024, 850, 709, 688, 647, 618 cm^–1^. HRMS (ESI+): *m*/*z* calcd for [C_14_H_12_O_2_ + H^+^]: 213.0910, found: 213.0920. m.p.: 196.5 °C–200.6 °C.

#### ((3-Bromopropoxy)­methyl)­ferrocene (**9**)

It was prepared according to the published procedure.^31^ However, the yield was increased to nearly quantitative (99%) by using 3-bromopropan-1-ol that was purified by column chromatography with a hexane/EtOAc (4:1, v/v) mixture as the mobile phase. Moreover, the purification of the final product was shortened to single-column chromatography with a hexane/EtOAc (39:1, v/v) mixture as the mobile phase, compared to three columns reported in the reference. Depending on the vendor, the use of commercial 3-bromopropan-1-ol directly without purification yielded 20–30% or even did not provide any reaction at all. ^1^H NMR (600 MHz, CDCl_3_, 25 °C): δ 4.29 (s, 2H), 4.23 (t, *J* = 1.8 Hz, 2H), 4.15 (t, *J* = 1.8 Hz, 2H), 4.13 (s, 5H), 3.55 (t, *J* = 5.9 Hz, 2H), 3.49 (t, *J* = 6.5 Hz, 2H), 2.09–2.06 (m, 2H). ^13^C NMR (151 MHz, CDCl_3_, 25 °C) δ 83.56, 69.52, 69.45, 68.61, 68.58, 67.45, 33.05, 30.93. The spectroscopic data correspond to the literature.

#### ((3-Iodopropoxy)­methyl)­ferrocene (**10**)

NaI (1.63 g, 10.9 mmol, 4.2 equiv) was added to a flask with **9** (0.871 g, 2.58 mmol, 1.0 equiv). The starting material was dissolved in acetone and refluxed (temperature of the heating block = 58 °C) for 1 h. Acetone was evaporated on a rotary evaporator. Et_2_O (100 mL) and water (100 mL) were added to the residue. The organic phase was separated, and the aqueous phase was extracted with additional Et_2_O (2 × 100 mL). Combined organic layers were washed with water (3 × 100 mL), dried over anhydrous Na_2_SO_4_, filtered, and evaporated. Yield: 94% (0.931 g) of a yellow dense liquid. ^1^H NMR (600 MHz, CDCl_3_, 25 °C): δ 4.29 (s, 2H), 4.23 (t, *J* = 1.9 Hz, 2H), 4.15 (t, *J* = 1.9 Hz, 2H), 4.14 (s, 5H), 3.49 (t, *J* = 5.9 Hz, 2H), 3.26 (t, *J* = 6.8 Hz, 2H), 2.06–1.99 (m, 2H). ^13^C NMR (126 MHz, CDCl_3_, 25 °C) δ 83.51, 69.51, 69.48, 69.33, 68.62, 68.57, 33.59, 3.83. IR-ATR ν̃ 3093 (Ar CH), 2853 (CH_2_), 1235, 1180, 1104, 1093, 1038, 1000, 818, 652, 641, 615 cm^–1^. HRMS (ESI+): *m*/*z* calcd for [C_14_H_17_FeIO + H^+^]:383.9668, found: 383.9673.

#### 3-(Ferrocenylmethoxy)­propyl) Methyl Viologen Hexafluorophosphate (**FcMV**)

Monomethyl viologen iodide (0.356 g, 1.19 mmol, 1.0 equiv, compound **11**) was added into a flask with **10** (0.917 g, 2.39 mmol, 2.0 equiv). The flask was evacuated and flushed with argon. The starting material was dissolved in anhydrous MeCN (20 mL) and refluxed (temperature of the heating block = 85 °C) for ca. 18 h under an inert atmosphere. After cooling to rt, the dark orange crystals were collected by filtration and washed with MeCN (3 × 5 mL). Yield 30% (0.245 g). The iodide salt (0.245 g, 0.359 mmol, 1.0 equiv) was dissolved in hot water (20 mL) and filtered through cotton, which was washed with additional water (3 × 1 mL). Into this solution, NH_4_PF_6_ (0.187 mg, 1.15 mmol, 3.2 equiv) in water (2 mL) was added, and the product precipitated. The pale orange precipitate was collected by filtration and washed with water (4 × ca. 5 mL). The product was dried in vacuo at 60 °C. Yield 79% (0.203 g). Total yield over both steps: 24%. ^1^H NMR (600 MHz, CD_3_CN, 25 °C): δ 8.83 (d, *J* = 6.9 Hz, 2H), 8.77 (d, *J* = 7.1 Hz, 2H), 8.29 (d, *J* = 7.0 Hz, 2H), 8.22 (d, *J* = 7.0 Hz, 2H), 4.66 (t, *J* = 6.5 Hz, 2H), 4.40 (s, 3H), 4.17 (s, 2H), 4.12 (s, 2H), 4.11 (s, 5H), 4.07 (s, 2H), 3.48 (t, *J* = 5.6 Hz, 2H), 2.28–2.17 (m, 2H). ^13^C NMR (151 MHz, CD_3_CN, 25 °C): δ 150.47, 147.47, 146.80, 127.70, 127.60, 70.70, 69.80, 69.53, 69.41, 66.61, 61.28, 49.62, 31.19. ^19^F NMR (565 MHz, CD_3_CN, 25 °C) δ −72.71 (d, *J* = 706.7 Hz). IR ν̃ 1716, 1644, 1568, 1509, 1455, 1340, 1083, 1050, 823 (PF_6_^–^), 742, 649, 624 cm^–1^. HRMS (ESI+): *m*/*z* calcd for [C_25_H_28_FeN_2_O^•+^]: 428.1540, found: 428.1549; HRMS (ESI-): *m*/*z* calcd for [PF_6_^–^]:144.9647, found: 144.9645. m.p.: 177.2 °C–189.4 °C.

#### *N*,*N*′-Dimethylviologen Hexafluorophosphate (**MV**)

A flask with bipyridyl (2.08 g, 13.3 mmol, 1.0 equiv) was evacuated and flushed with argon. Anhydrous MeCN (20 mL) and MeI (4.10 mL, 66.6 mmol, 5.0 equiv) were added. The reaction mixture was refluxed (temperature of the heating block = 85 °C) for ca. 18 h under an inert atmosphere. After cooling to rt, the red precipitate was collected by filtration and washed with MeCN (4 × 5 mL). Yield: 99% (6.79 g). The iodide salt (0.951 g, 2.16 mmol, 1.0 equiv) was dissolved in hot water (10 mL) and filtered through cotton, which was washed with additional water (2 × 1 mL). Into this solution, NH_4_PF_6_ (1.06 mg, 6.53 mmol, 3.0 equiv) in water (2 mL) was added, and the product precipitated. The pale-yellow precipitate was collected by filtration and washed with water (3 × ca. 5 mL). The product was dried in vacuo at 60 °C. Yield 85% (0.874 g). Total yield over both steps: 84%. ^1^H NMR (600 MHz, CD_3_CN, 25 °C): δ 8.85 (d, *J* = 6.8 Hz, 4H), 8.37 (d, *J* = 6.2 Hz, 4H), 4.40 (s, 6H). ^13^C NMR (151 MHz, CD_3_CN, 25 °C): δ 150.64, 147.48, 127.81, 49.61. ^19^F NMR (565 MHz, CD_3_CN, 25 °C) δ −72.11, −73.36. The spectroscopic data correspond to the literature.^40^

### HPLC

HPLC analysis was performed on an LC20 chromatograph (Shimadzu, Kyoto, Japan), composed of a DGU-20A3 solvent degasser, two LC-20AD binary gradient pumps, a SIL-20AC autosampler with a 500 μL sample loop, a CTO-20AC column oven, an SPD-M20A photodiode array detector (PDA), and a CBM-20A system controller. All samples were analyzed using a column Luna Omega C18 (100 × 3 mm, 5 μm) at 40 °C with a flow rate of 1 mL/min. The mobile phase was 95 vol % MeCN in water (isocratic). Absorption at three different wavelengths (260, 369, and 633 nm) was monitored. The purity was established from the integrals of the product peak and the sum of all present peaks in the chromatogram (Figures S20–22). The purities of all samples were comparable, corresponding to 94–95%.

### Fluorescence Measurements

Lifetimes were determined on an FLS-1000 (Edinburgh Instruments, Livingston, UK) with a diode laser HPL-655 (λ_ex_ = 653.9 nm; 50 ns pulse period). Fluorescence quantum yields (Φ_*F*_) were determined by the comparative method^41^ using zinc­(II) phthalocyanine as the reference (Φ_F,ref_ = 0.32)^42^ in THF. The excitation wavelength was 602 nm. The values were calculated according to eq 8, where *I* and *I*_ref_ are the integrated intensities of the fluorescence of the sample and reference, respectively, OD and OD_ref_ are the optical densities of the sample and reference, respectively, and *n* and *n*_ref_ are the refractive indices of the solvent used for the sample (MeCN) and for the reference (THF), respectively. The measurements were performed in triplicate; the presented data are the mean value of the determined Φ_*F*_. The estimated experimental error was below 5%. Absorption of the samples at the excitation wavelength was kept below 0.05 and at a Q-band maximum below 0.1 to avoid the inner-filter effect. The results of Φ_*F*_ were corrected for the refractive indices of the solvents.

8 $$\Phi_{F}=\Phi_{F,ref}\frac{I}{I_{ref}}\frac{OD_{ref}}{OD}\frac{n^{2}}{n_{ref}^{2}}$$

### Fluorescence Titrations

A solution of AzaPc (*c* = 5.00 μM) was prepared in MeCN. Samples of **FcMV**, **Fc**, and **MV** were dissolved in this solution to get a 200 mM concentration of the quencher. The solution of AzaPc is used to maintain the concentration of AzaPc constant during the titrations. Fluorescence emission spectra and lifetimes of the samples of AzaPc were recorded first without any quencher and then after stepwise addition of the quencher. The solution was stirred in the cuvette for 2 min between mixing and measurement. The data were treated according to published methodology to calculate quenching and association constants.^33^

### Cyclic Voltammetry

The electrochemical measurements (cyclic voltammetry and SWV) were performed at room temperature (25 °C) using an Autolab PGSTAT101 potentiostat. Measurements were carried out with a three-electrode setup consisting of a Pt working electrode, a Pt counter electrode, and a Ag/AgCl reference electrode separated from the bulk solution by an integrated salt bridge. The detailed procedure was as follows: 0.1 M solution of tetrabutylammonium hexafluorophosphate in pyridine (4 mL) as a supporting electrolyte was added to the cell and bubbled with argon for 5 min to remove oxygen, and blank was measured. Afterward, the corresponding compound (5–10 mg) was added, and the solution was bubbled for another 5 min. Half-wave potentials (*E*_1/2_) were obtained from SWV with a potential step 5 mV and a scan rate of 100 mV/s. The obtained data listed in Table 2 were referenced to SCE with ferrocene as the internal standard (*E*_1/2_ (Fc/Fc^+^) = 0.56 V vs SCE^34^).

### Time-Resolved Absorption Spectroscopy

Transient absorption experiments were carried out using a commercially available apparatus from Ultrafast Systems. Briefly, a ytterbium femtosecond laser was used to generate 1030 nm with 400 μJ pulses at 1 kHz. The output was split into two parts, where 75% of the 1030 nm pulses were used to pump a collinear Optical Parametric Amplifier (OPA, APOLLO-Y) tuned to pump 640 nm pulses (ca. 250 fs, 80–200 nJ at the sample position) for sample excitation, and 25% were used for generation of supercontinuum white light probe pulses by focusing into one of two different crystals, giving a probe spectrum ranging from 350 to 500 or 500 to 950 nm. Probe pulses were delayed via an optical motorized delay line with an 8 ns time window. The pump and probe pulses were focused colinearly into the sample to spot sizes of ca. 400 and 150 μm full width at half-maximum, respectively. A spectrometer with a linear array CMOS sensor (1024 pixels) was used for detection. The isotropic spectral signals were secured by the use of an achromatic broadband depolarizer for the pump (ThorLabs, DPP-25-A). The sample in the cuvette with a 2 mm path length was randomly moved at 1 mm/s speed through the measurement. The stability of the sample was verified by recording steady-state absorption spectra before and after each measurement. The data were corrected for cross-phase modulation and globally analyzed in OPTIMUS software^43^ using a sequential model. The presented lifetimes have a standard deviation of ±5%.

## Supplementary Material



## Data Availability

The data underlying this study are openly available in Zenodo at: https://doi.org/10.5281/zenodo.17485795.

## Notes

Characterization of the synthesized compounds, including NMR, HRMS, and IR spectra, HPLC analyses, and photophysical characterization; voltammograms from CV and SWV; additional graphs from TAS; details for fluorescence titration experiments; and TLCs from the AzaPc syntheses (PDF)
