# Supplementary material for: Enhanced Quenching in an Azaphthalocyanine–Ferrocene Supramolecular Dyad upon Charge-Transfer Complex Formation
Source: Inorg Chem. 2025 Dec 15;64(51):25077–88. doi: 10.1021/acs.inorgchem.5c03733 (PMC12754792; doi:10.1021/acs.inorgchem.5c03733)
Supplement: Supplementary file 1 [file ic5c03733_si_001.pdf]

Supporting Information

Enhanced Quenching in an Azaphthalocyanine-Ferrocene Supramolecular Dyad upon Charge-Transfer Complex Formation

*Jana Lapesova,[1] Jiri Demuth,[1] Veronika Novakova,[1] Lucie Ludvikova,[2] Tomas Slanina,[2] Petr Zimcik[1]**

[1]Department of Pharmaceutical Chemistry and Pharmaceutical Analysis, Faculty of Pharmacy in Hradec Kralove, Charles University, Akademika Heyrovskeho 1203, 500 05 Hradec Kralove, Czech Republic; [2]Institute of Organic Chemistry and Biochemistry of the Czech Academy of Sciences, Flemingovo namesti 542/2, 160 00 Prague 6, Czech Republic

Email: zimcik@faf.cuni.cz

Supporting Information

Contents

1. Characterization 3
   1.1. NMR spectra 3
   1.2. HRMS Spectra 10
   1.3. IR Spectra of AzaPcs **1** and **6** 14
   1.4. HPLC Analyses 15
   1.5. Photophysical Characterization 18
2. Fluorescence Titrations 19
   2.1. Fluorescence Spectra of **2** and Stern-Volmer Plot 19
   2.2. Stern-Volmer plots for **Fc** and **MV** 19
   2.3. Lifetimes (AzaPc **1**) 20
   2.4. Calculations 21
3. TLCs 22
4. Electrochemistry 23
5. Transient Absorption Spectroscopy 24
6. References 24

1. Characterization

1.1. NMR spectra

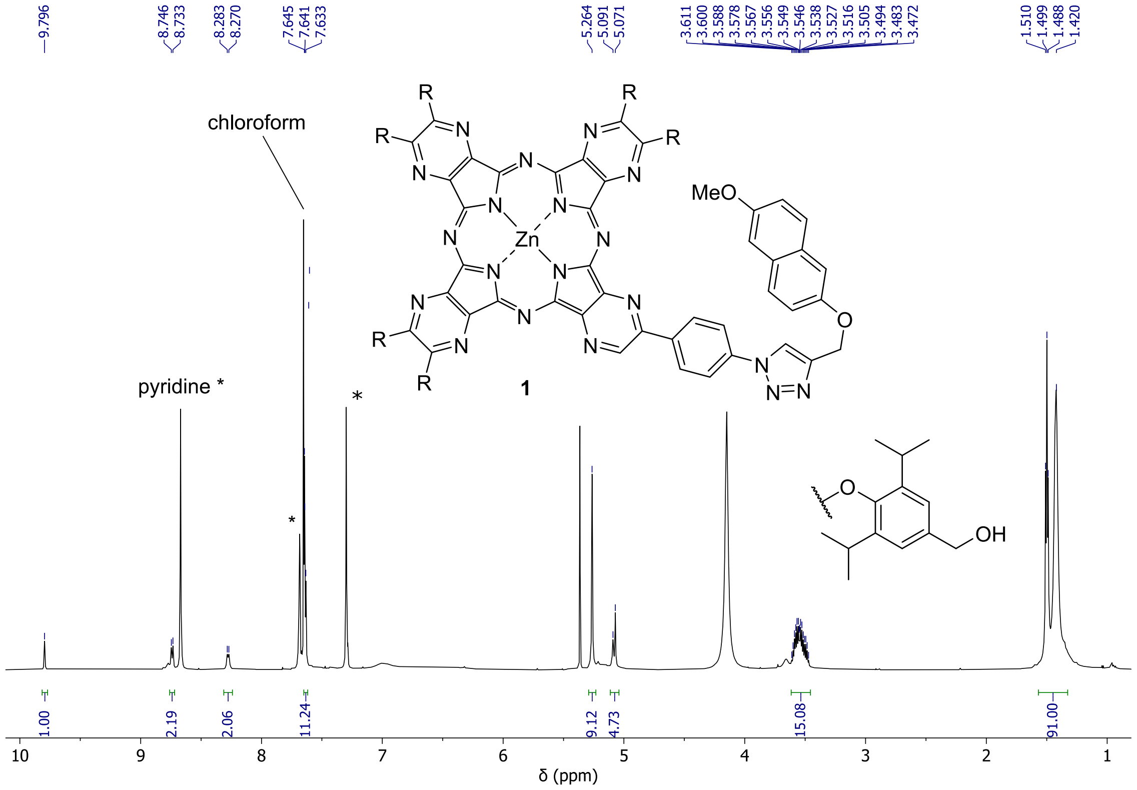

Figure S1. ^{1}H NMR (600 MHz, $CDCl_3$ and pyridine-d_5 (3:1, v/v), 25 °C) spectrum of **1**.

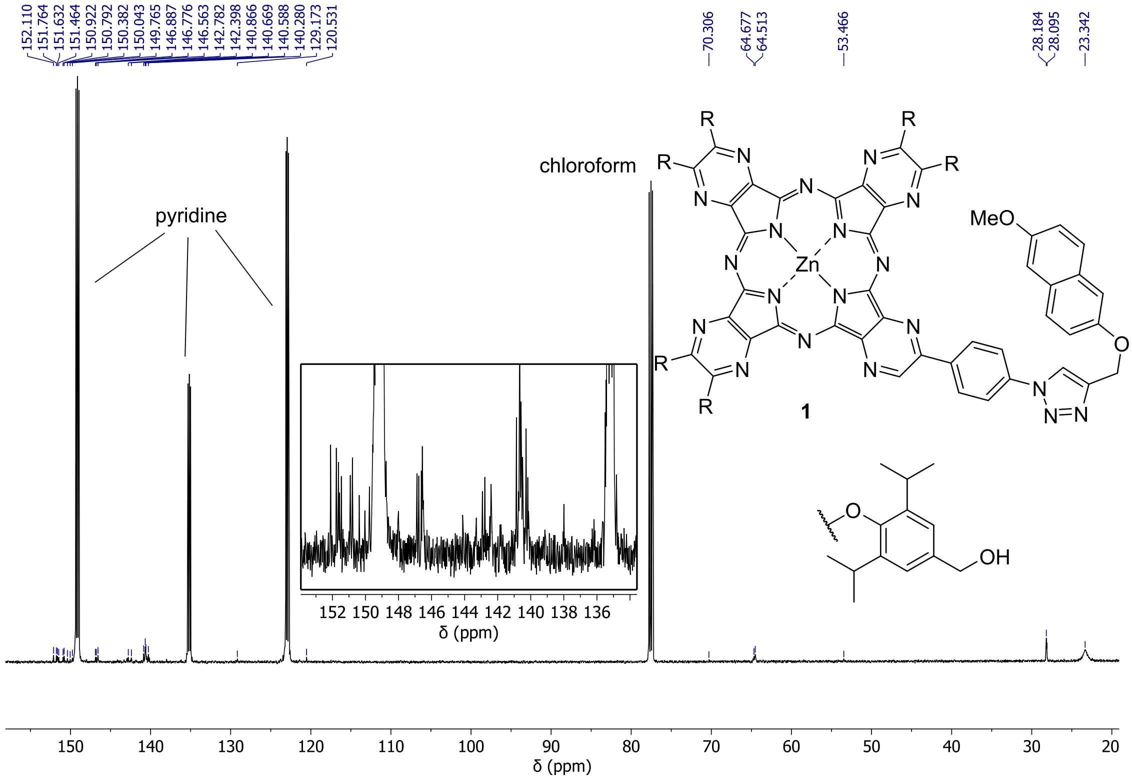

Figure S2. ^{13}C NMR (151 MHz, $CDCl_3$ and pyridine-d_5 (3:1, v/v), 25 °C) spectrum of **1**.

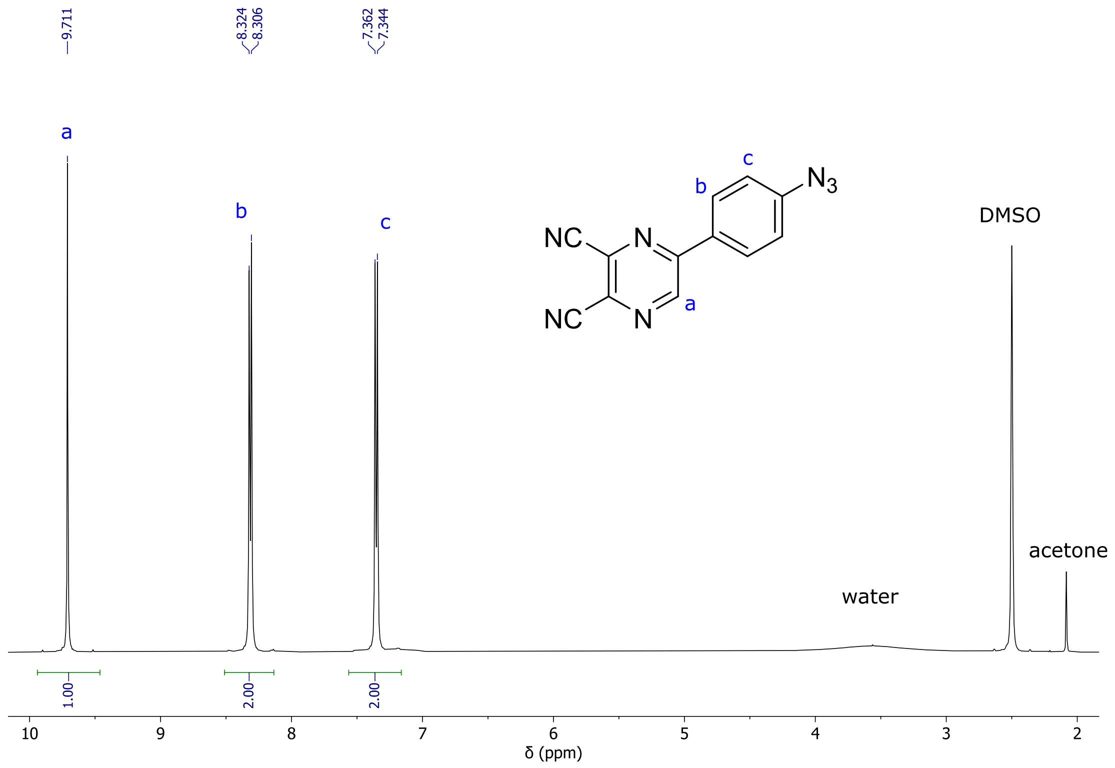

Figure S3. ^{1}H NMR (500 MHz, DMSO-d_6, 25 °C) spectrum of **4**.

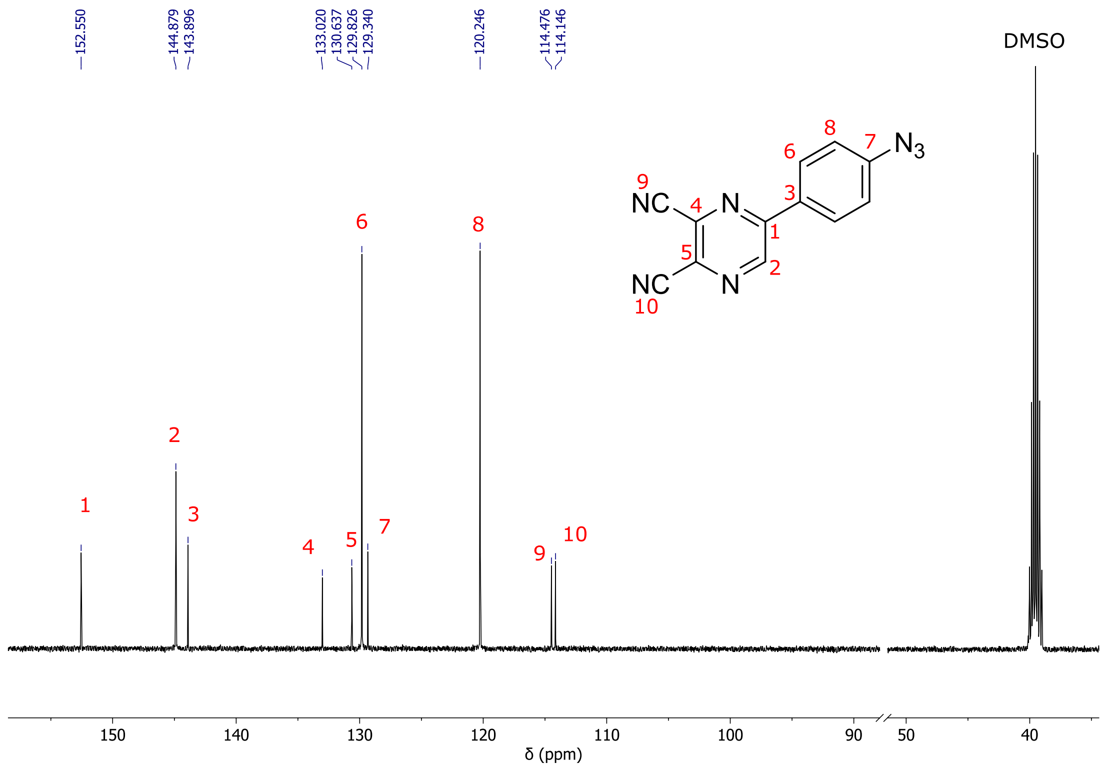

Figure S4. ^{13}C NMR (126 MHz, DMSO-d_6, 25 °C) spectrum of **4**.

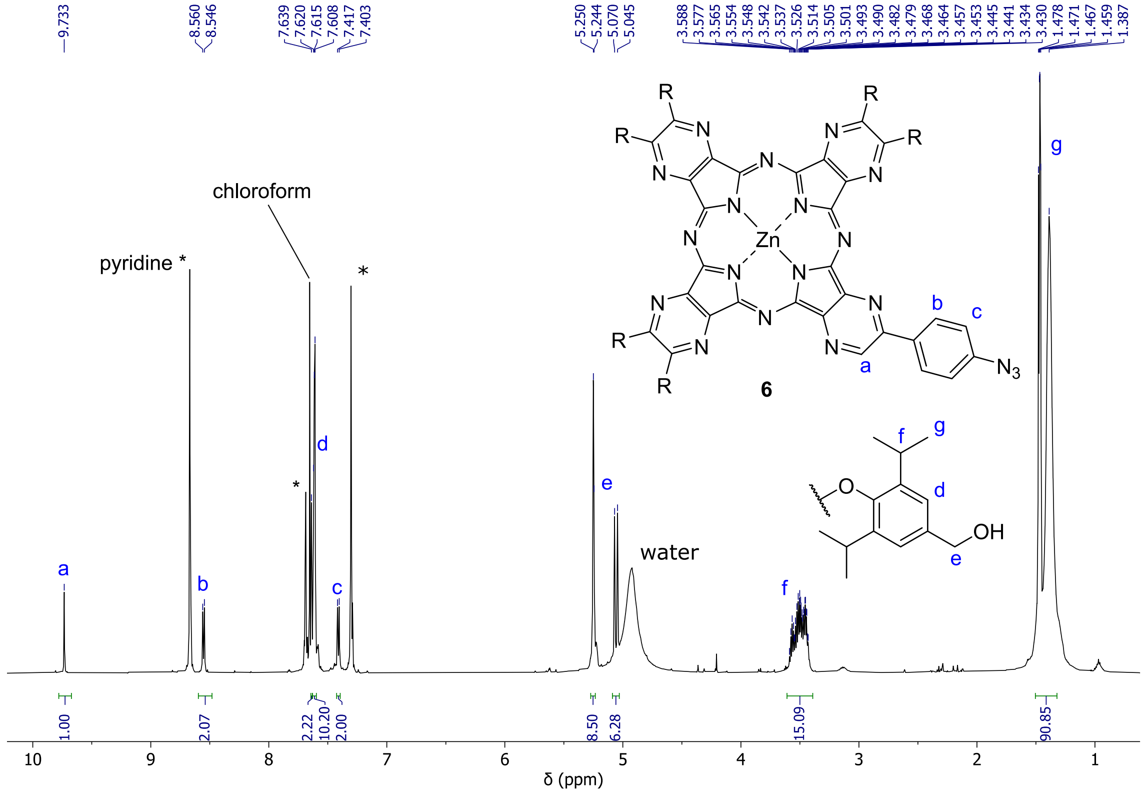

Figure S5. ^{1}H NMR (600 MHz, $CDCl_3$ and pyridine-d_5 (3:1, v/v), 25 °C) spectrum of **6**.

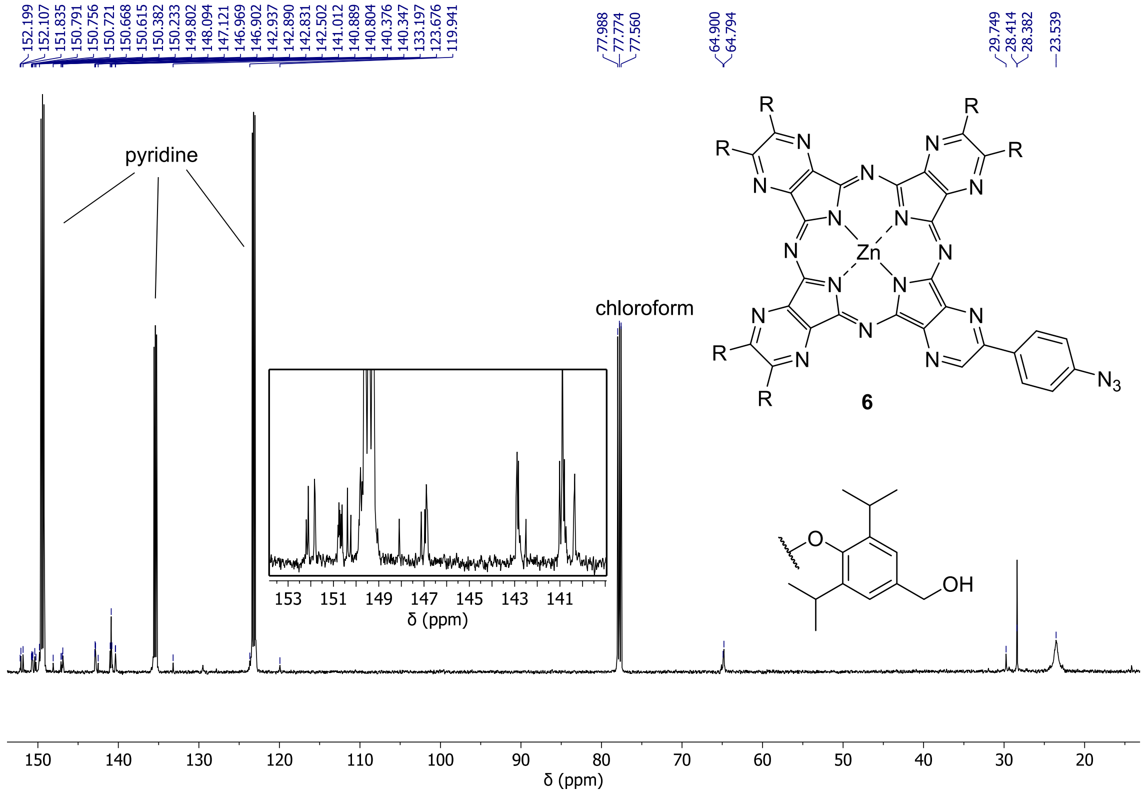

Figure S6. ^{13}C NMR (151 MHz, $CDCl_3$ and pyridine-d_5 (3:1, v/v), 25 °C) spectrum of **6**.

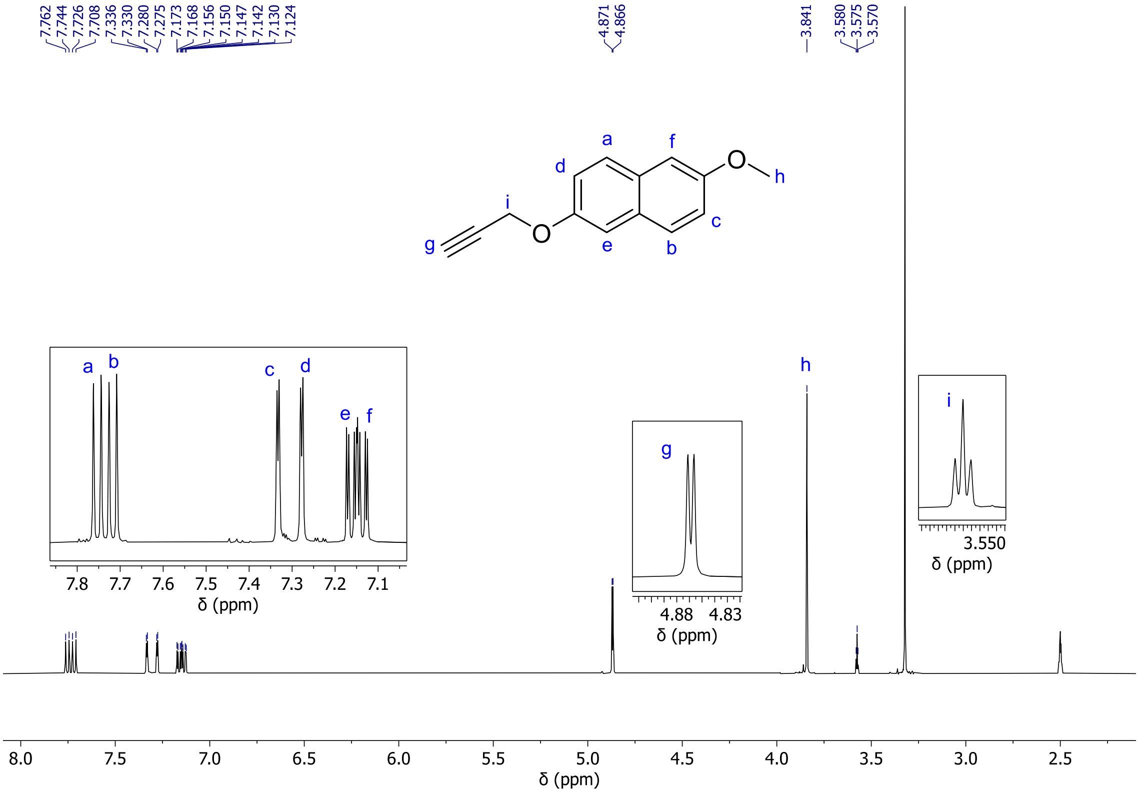

Figure S7. ^{1}H NMR (600 MHz, DMSO-d_6, 25 °C) spectrum of **7**.

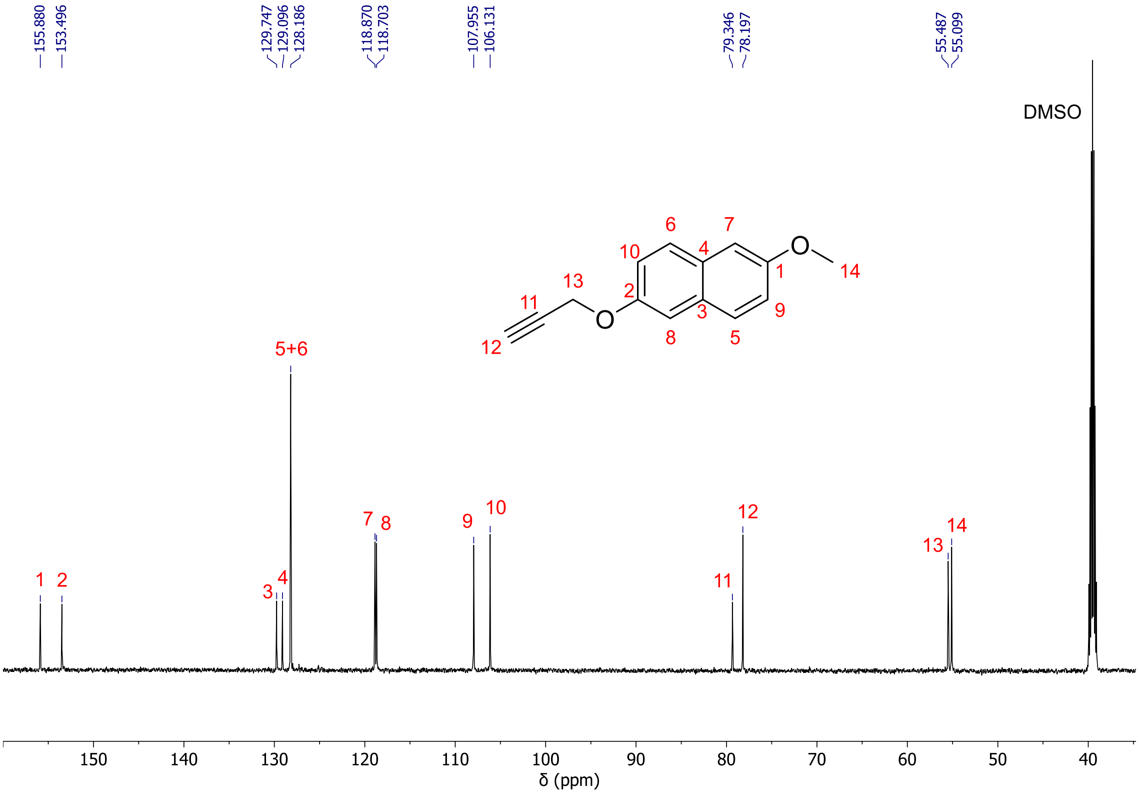

Figure S8. ^{13}C NMR (151 MHz, DMSO-d_6, 25 °C) spectrum of **7**.

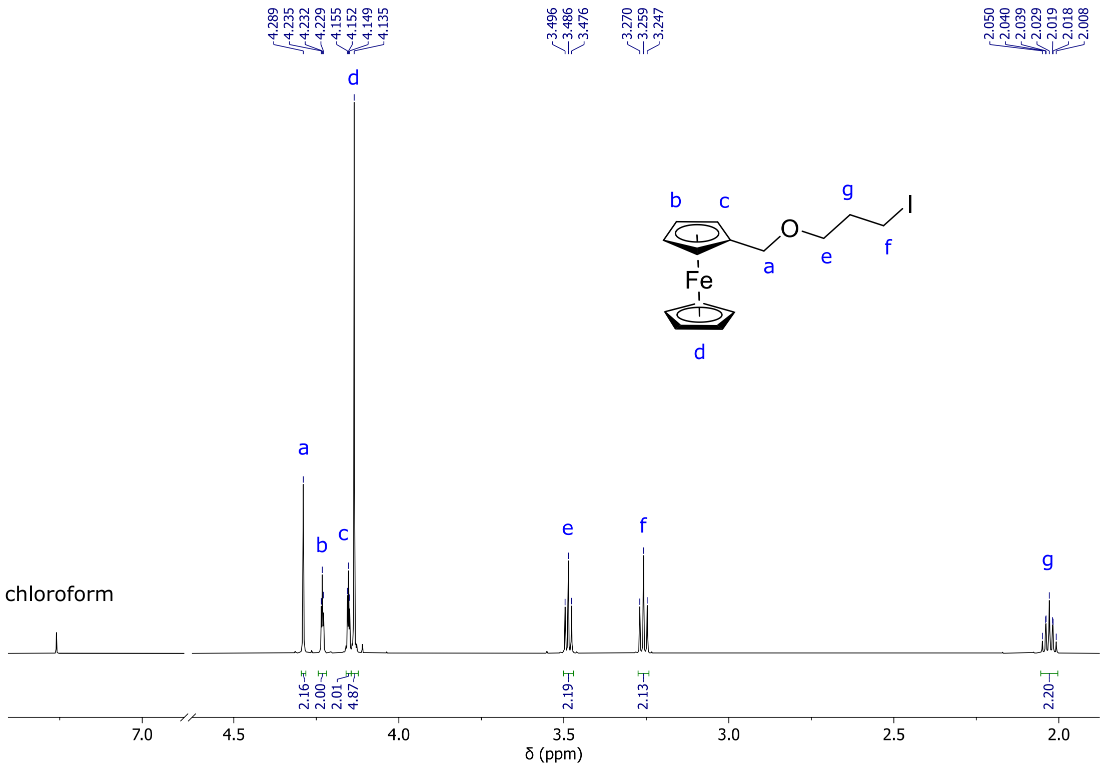

Figure S9. ^{1}H NMR (600 MHz, $CDCl_3$, 25 °C) spectrum of **10**.

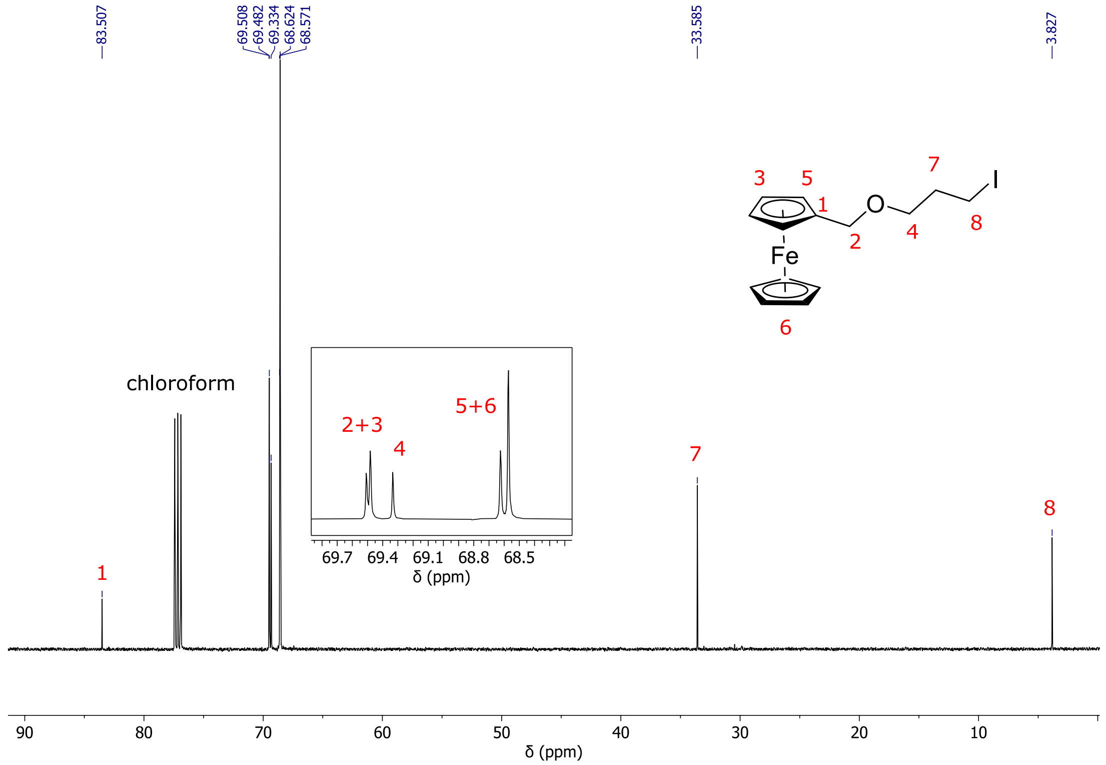

Figure S10. ^{13}C NMR (126 MHz, $CDCl_3$, 25 °C) spectrum of **10**.

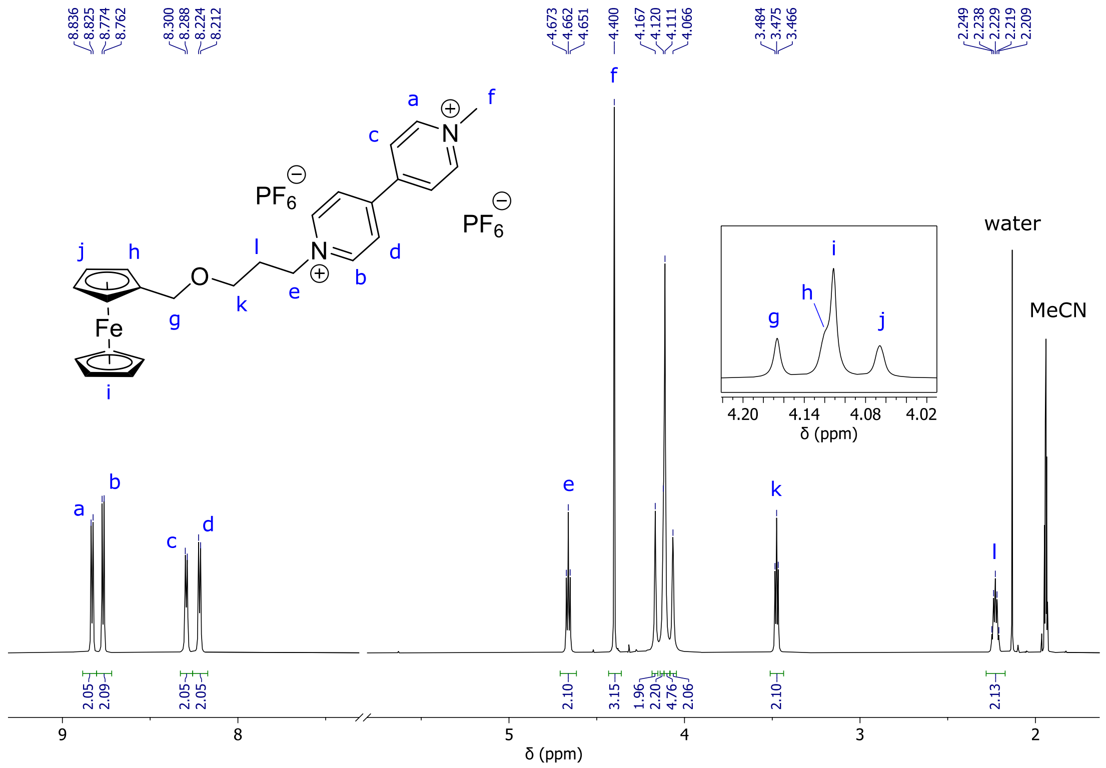

Figure S11. ^{1}H NMR (600 MHz, CD_3CN, 25 °C) spectrum of **FcMV**.

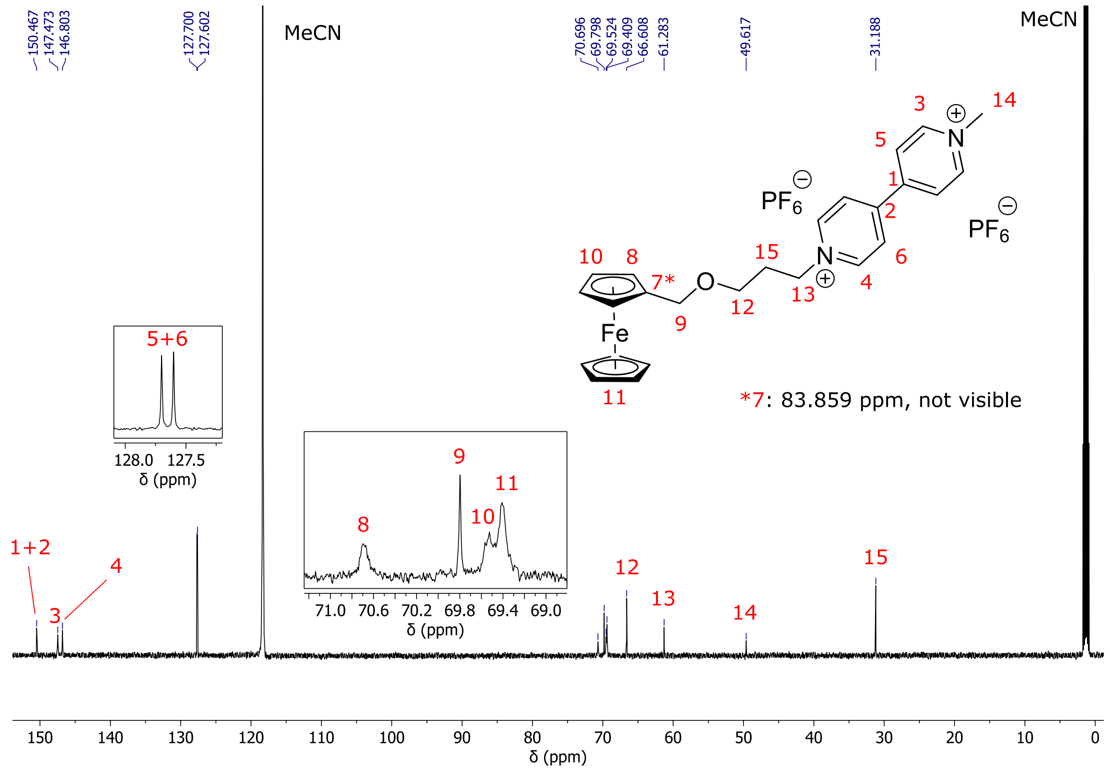

Figure S12. ^{13}C NMR (151 MHz, CD_3CN, 25 °C) spectrum of **FcMV**.

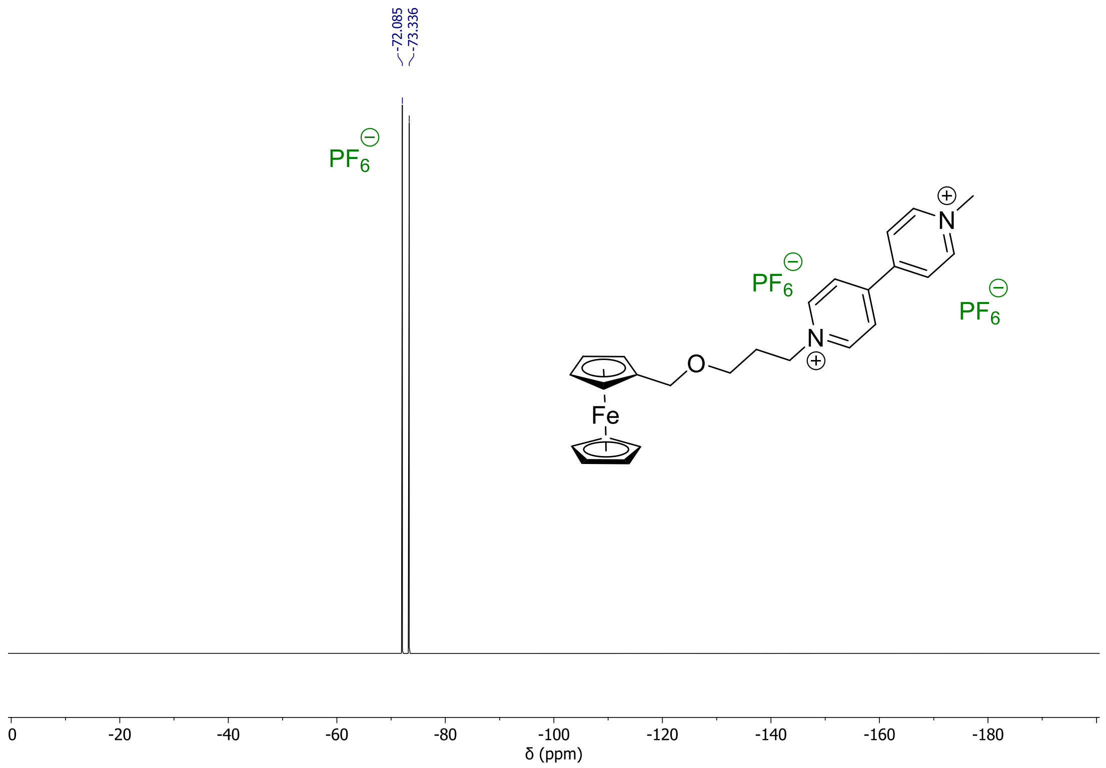

Figure S13. ^{19}F NMR (565 MHz, CD_3CN, 25 °C) spectrum of **FcMV**.

1.2. HRMS Spectra

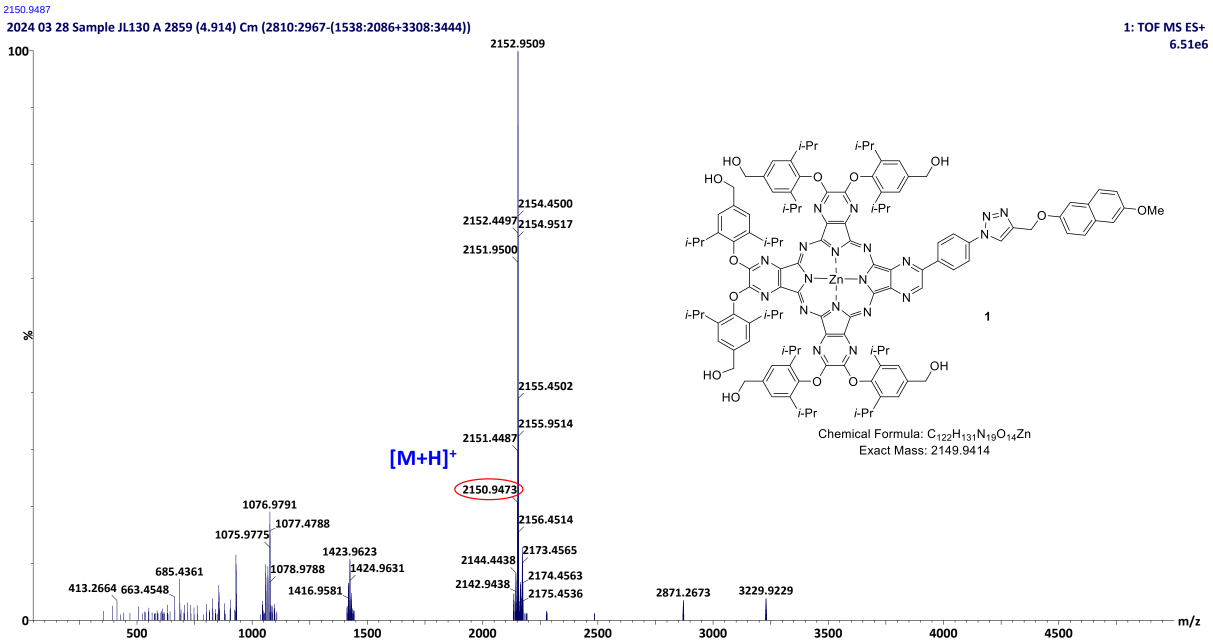

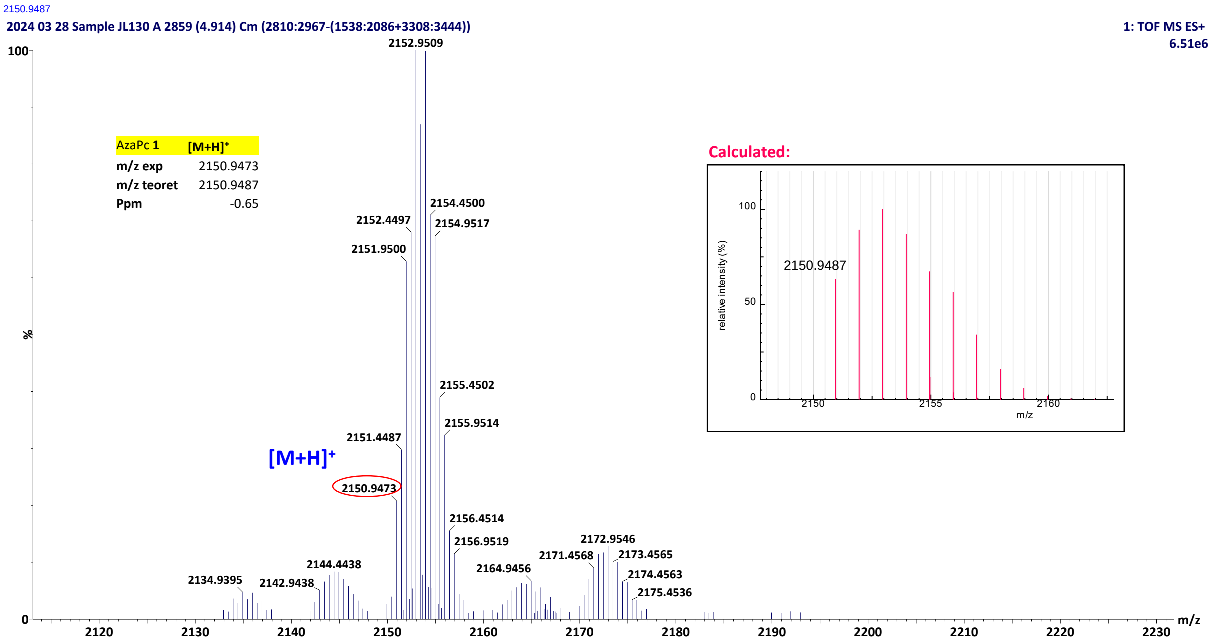

Figure S14. HRMS spectrum (ESI+) of **1**, inset: calculated pattern.[1]

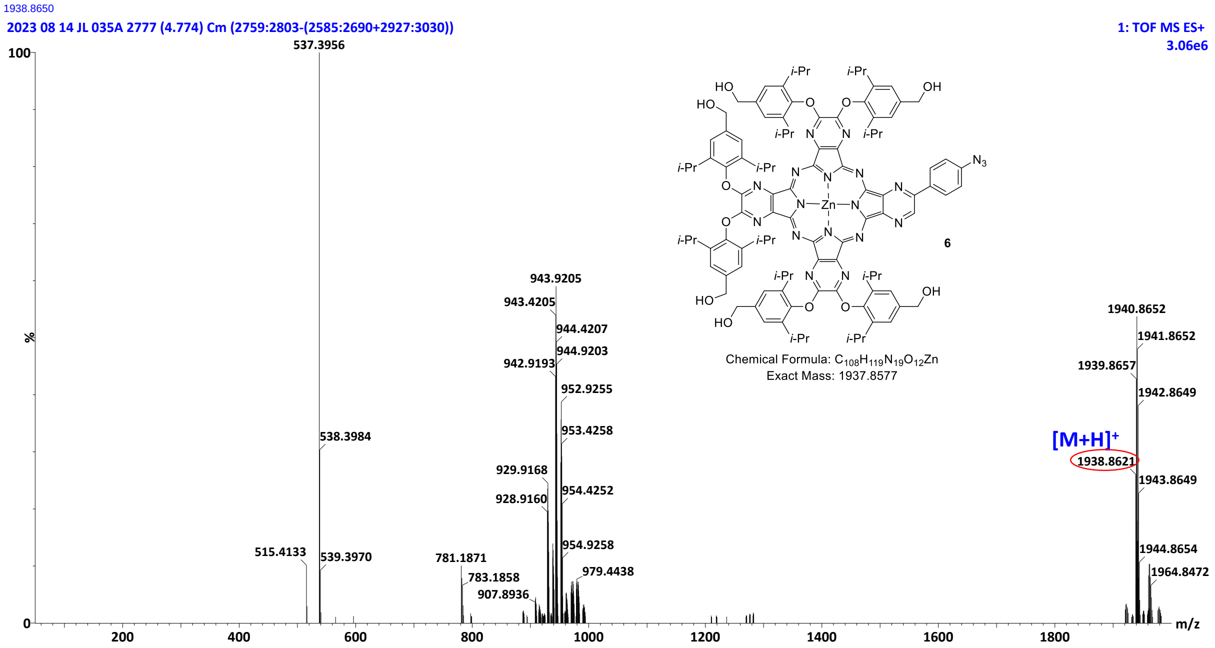

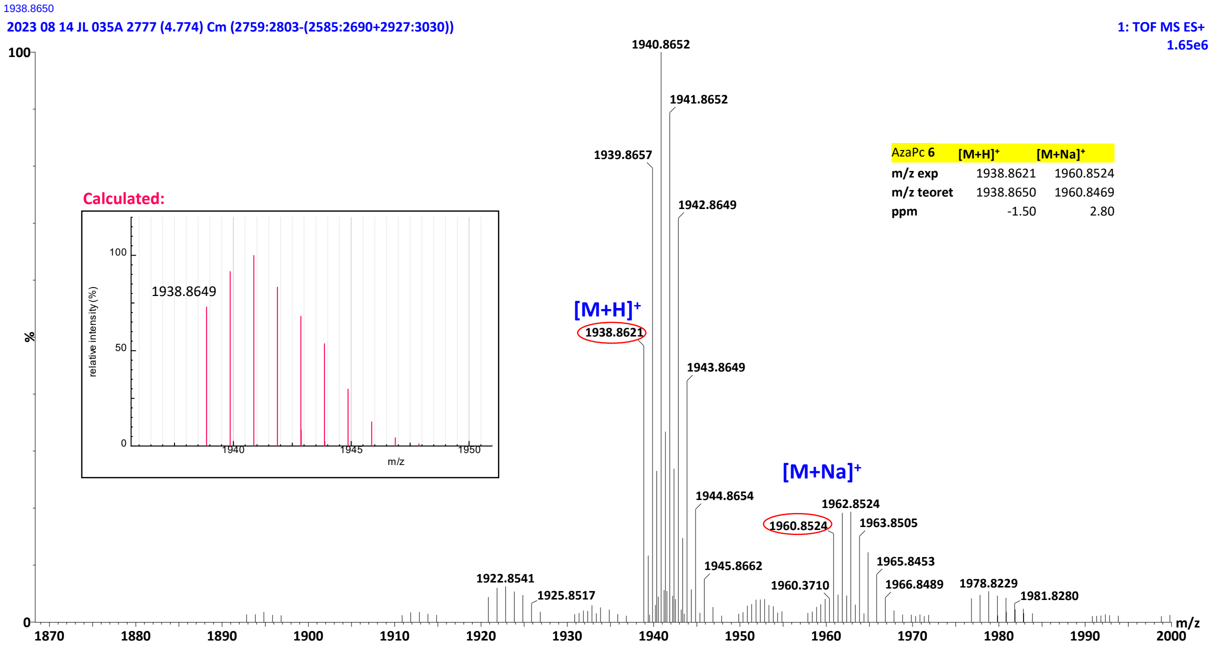

Figure S15. HRMS spectrum (ESI+) of **6**, inset: calculated pattern.[1]

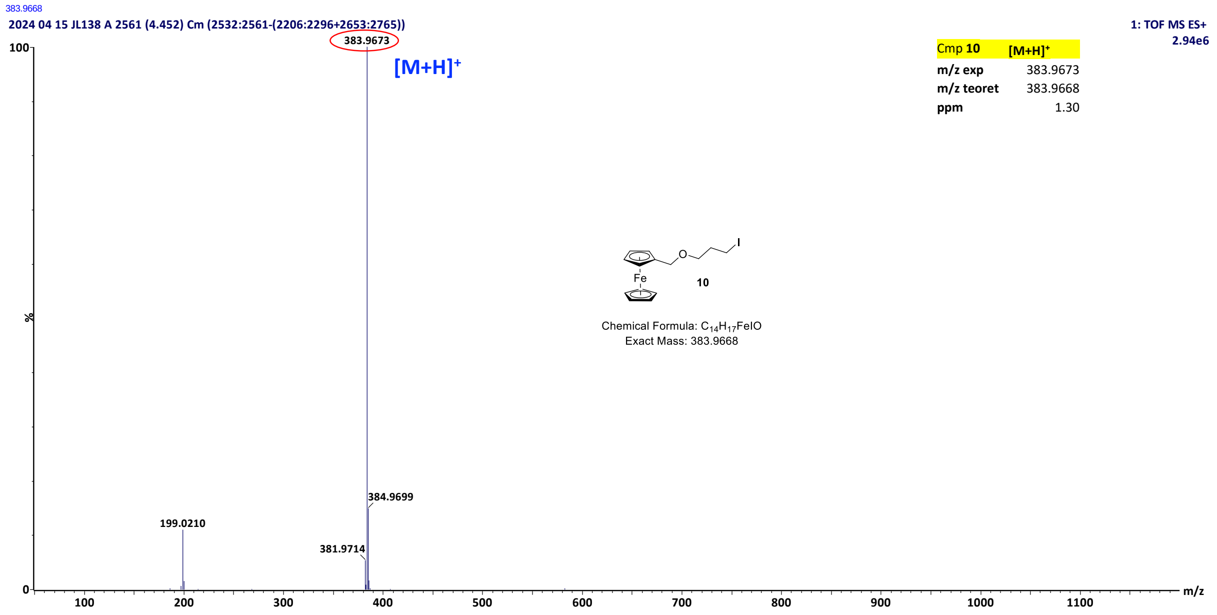

Figure S16. HRMS spectrum (ESI+) of **10**.

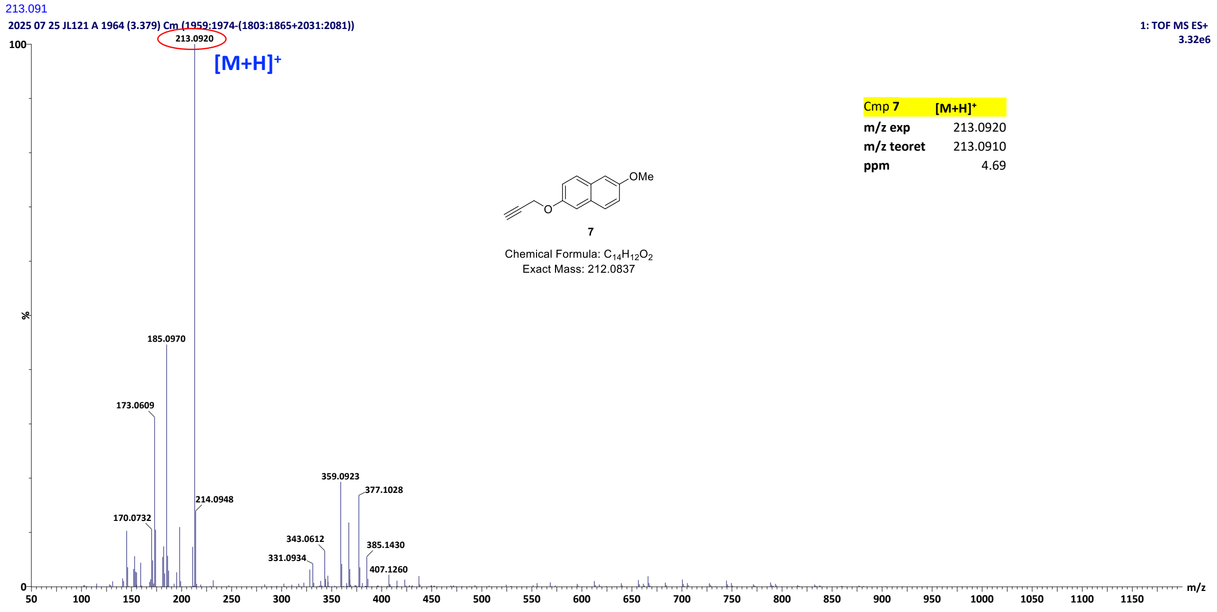

Figure S17. HRMS spectrum (ESI+) of **7**.

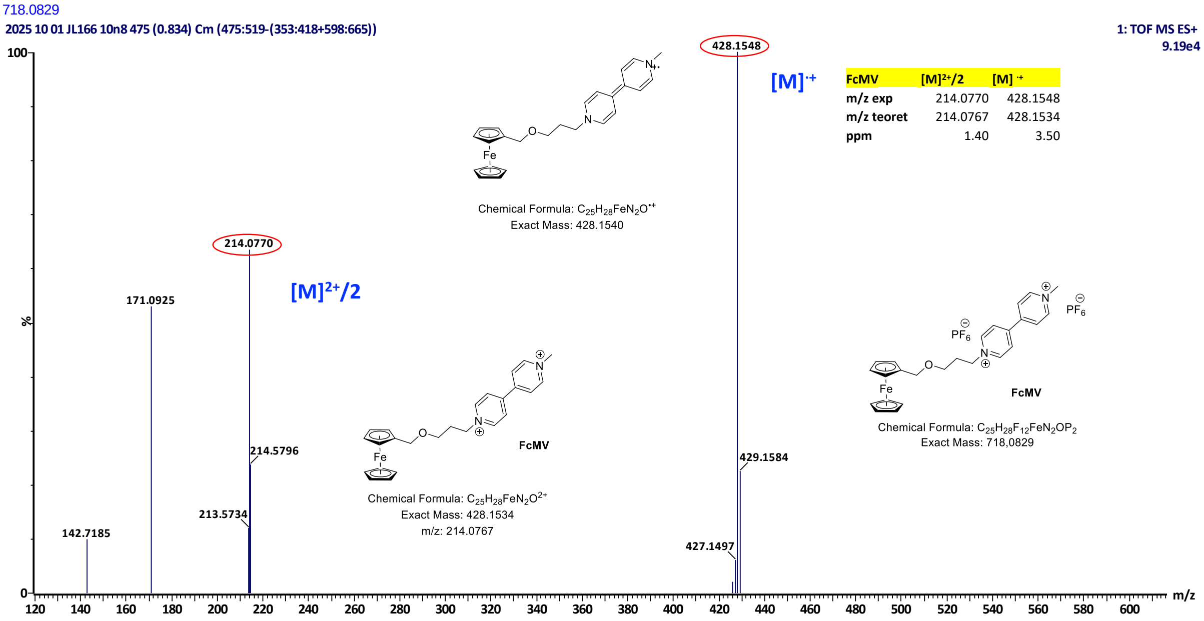

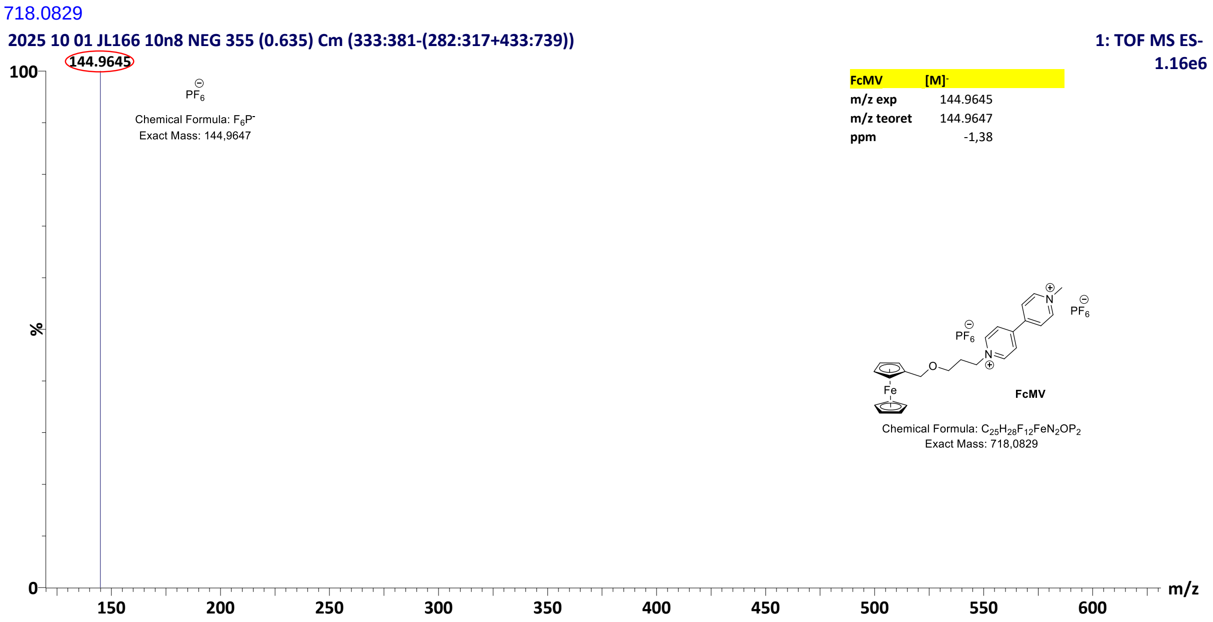

Figure S18. HRMS spectra (ESI+ and ESI-) of **FcMV**.

1.3. IR Spectra of AzaPcs **1** and **6**

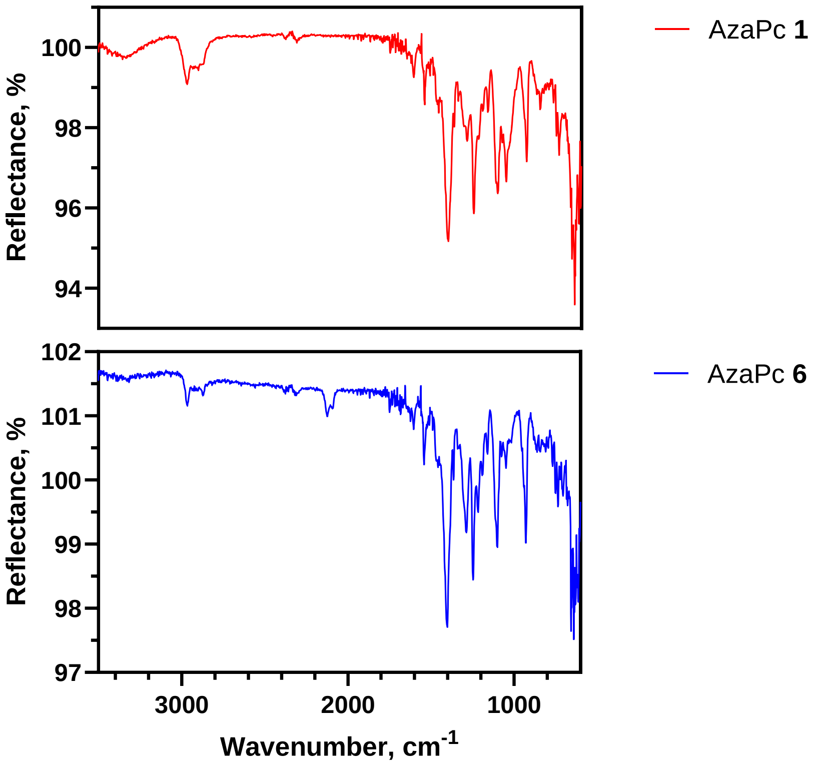

Figure S19. Comparison of IR-ATR spectra of AzaPcs **1** and **6**.

1.4. HPLC Analyses

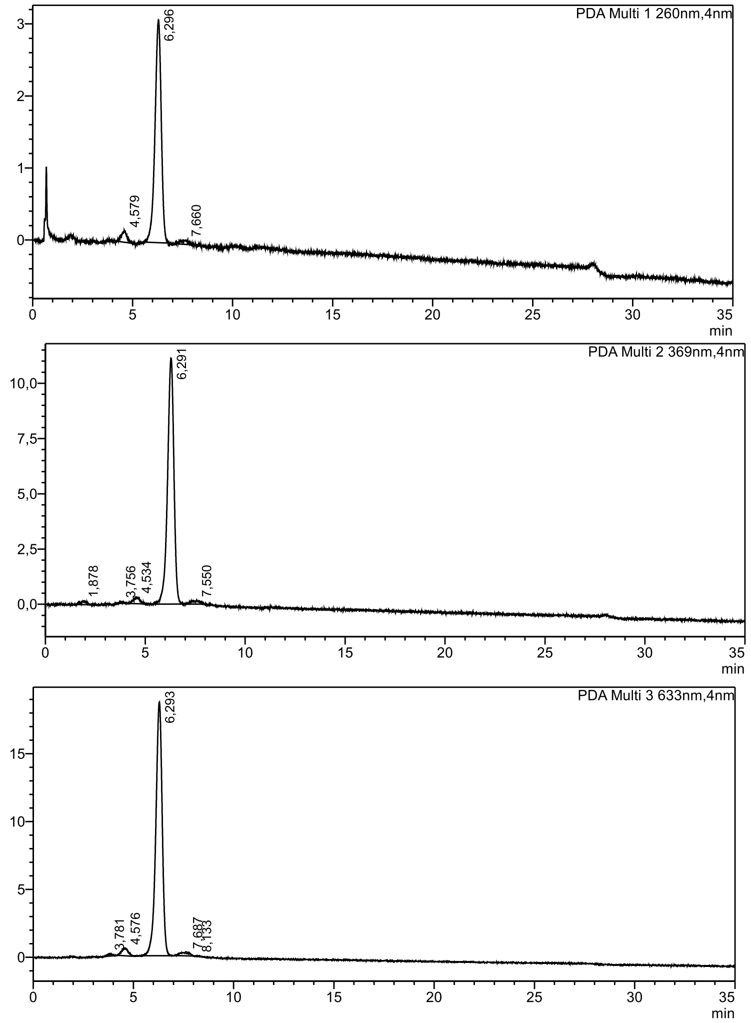

PDA Ch1 260nm

Peak#	Ret. Time	Area	Area%
1	4,579	3258	4,504
2	6,296	67567	93,388
3	7,660	1525	2,108
Total		72351	100,000

PDA Ch2 369nm

Peak#	Ret. Time	Area	Area%
1	1,878	3304	1,255
2	3,756	1064	0,404
3	4,534	6595	2,506
4	6,291	247486	94,021
5	7,550	4775	1,814
Total		263224	100,000

PDA Ch3 633nm

Peak#	Ret. Time	Area	Area%
1	3,781	2464	0,563
2	4,576	12683	2,896
3	6,293	413684	94,461
4	7,687	8560	1,955
5	8,133	549	0,125
Total		437941	100,000

Figure S20. Chromatograms of a sample of **1** analyzed by HPLC using 95 % MeCN in water and a table with integrals (areas under the curves) of each peak. The area of the corresponding peak at the retention time of 6.29 min expressed in % represents purity of the sample. Absorbance was monitored at 260 nm, 369 nm and 633 nm.

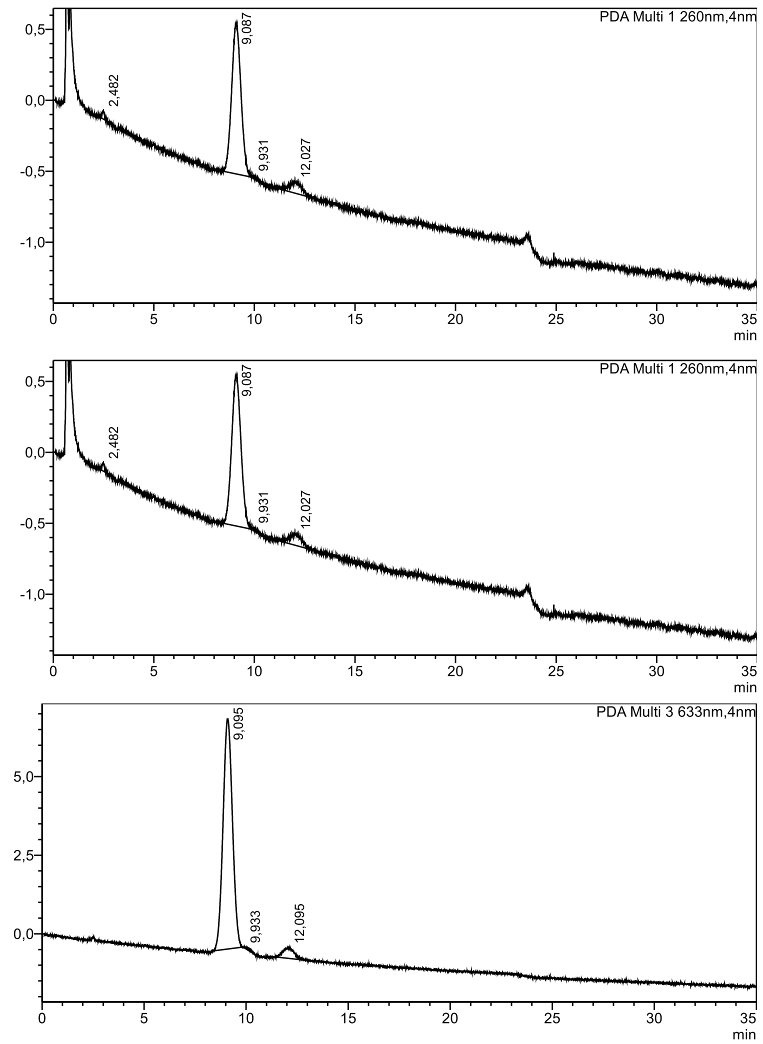

PDA Ch1 260nm

Peak#	Ret. Time	Area	Area%
1	2,482	489	1,267
2	9,087	34370	89,023
3	9,931	313	0,811
4	12,027	3436	8,899
Total		38607	100,000

PDA Ch2 369nm

Peak#	Ret. Time	Area	Area%
1	9,102	189851	94,841
2	9,864	597	0,298
3	12,075	9729	4,860
Total		200177	100,000

PDA Ch3 633nm

Peak#	Ret. Time	Area	Area%
1	9,095	235216	94,135
2	9,933	1277	0,511
3	12,095	13377	5,354
Total		249870	100,000

Figure S21. Chromatograms of a sample of **2** analyzed by HPLC using 95 % MeCN in water and a table with integrals (areas under the curves) of each peak. The area of the corresponding peak at the retention time of 9.10 min expressed in % represents purity of the sample. Absorbance was monitored at 260 nm, 369 nm and 633 nm.

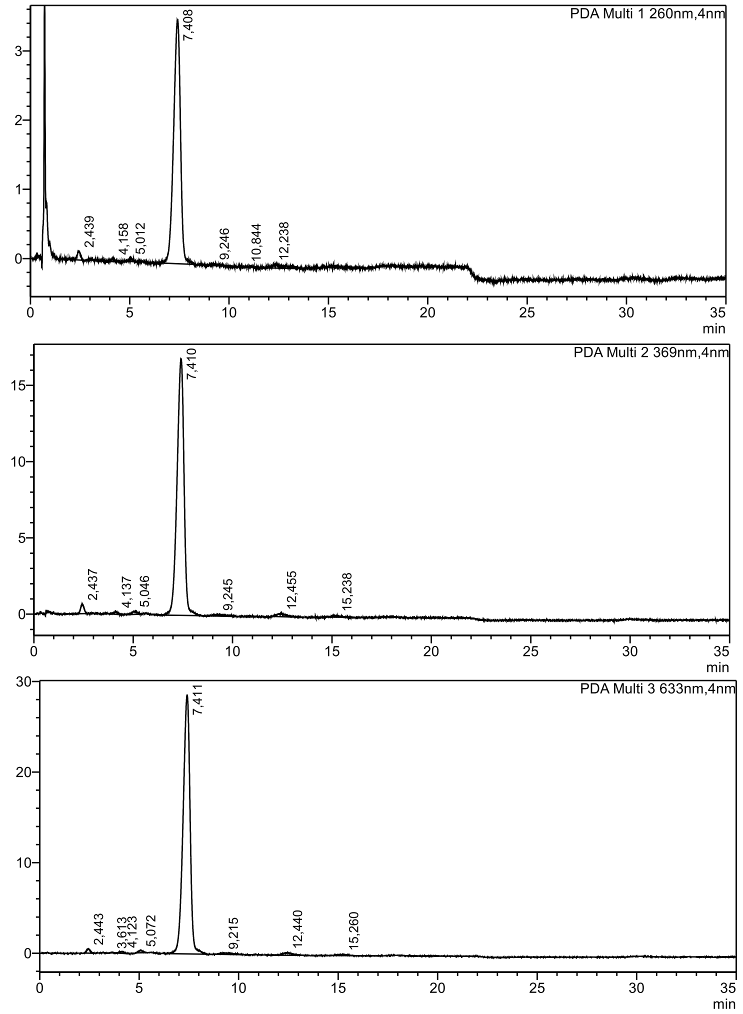

PDA Ch1 260nm

Peak#	Ret. Time	Area	Area%
1	2,439	1426	1,564
2	4,158	605	0,663
3	5,012	676	0,741
4	7,408	85179	93,378
5	9,246	540	0,592
6	10,844	248	0,272
7	12,238	2546	2,791
Total		91220	100,000

PDA Ch2 369nm

Peak#	Ret. Time	Area	Area%
1	2,437	7540	1,719
2	4,137	2521	0,575
3	5,046	4040	0,921
4	7,410	410219	93,533
5	9,245	4681	1,067
6	12,455	6639	1,514
7	15,238	2944	0,671
Total		438583	100,000

PDA Ch3 633nm

Peak#	Ret. Time	Area	Area%
1	2,443	5800	0,792
2	3,613	372	0,051
3	4,123	3123	0,427
4	5,072	3964	0,541
5	7,411	697049	95,209
6	9,215	7818	1,068
7	12,440	9923	1,355
8	15,260	4074	0,556
Total		732122	100,000

Figure S22. Chromatograms of a sample of **6** analyzed by HPLC using 95 % MeCN in water and a table with integrals (areas under the curves) of each peak. The area of the corresponding peak at the retention time of 7.41 min expressed in % represents purity of the sample. Absorbance was monitored at 260 nm, 369 nm and 633 nm.

1.5. Photophysical Characterization

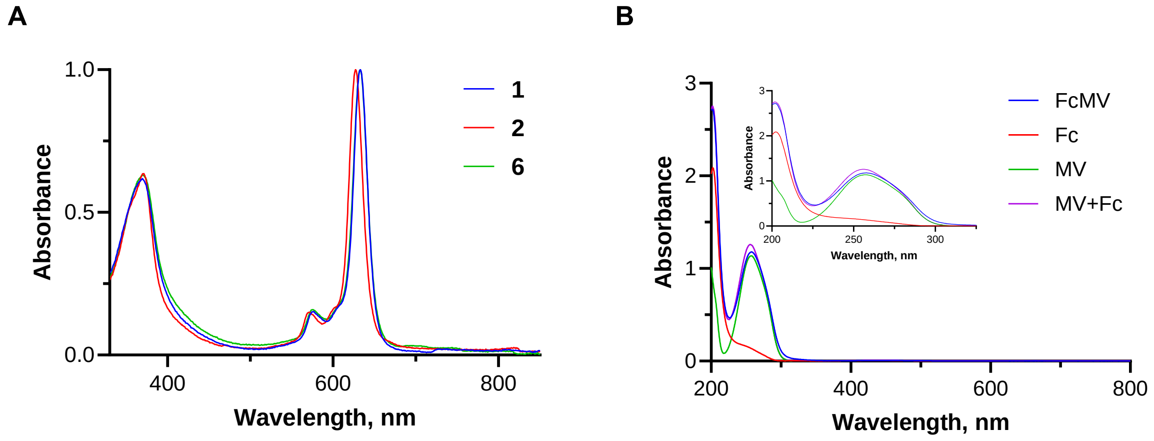

Figure S23. Absorption spectra of (A) AzaPcs **1**, **2** and **6** (1 µM, MeCN, normalized), and quenchers (B) **FcMV**, **Fc** and **MV** (50 µM, MeCN).

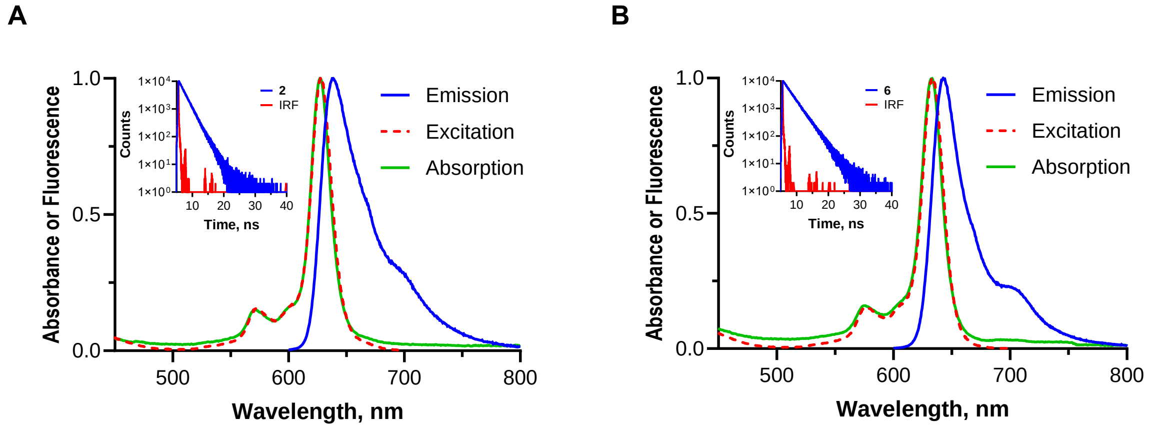

Figure S24. Normalized absorption (green line), excitation (dashed red line; emission was measured at 710 nm) and emission (blue; excitation at 590 nm) spectra of (A) **2** and (B) **6** (1 µM, MeCN). Insets show fluorescence decays during the lifetime measurement.

2. Fluorescence Titrations

2.1. Fluorescence Spectra of **2** and Stern-Volmer Plot

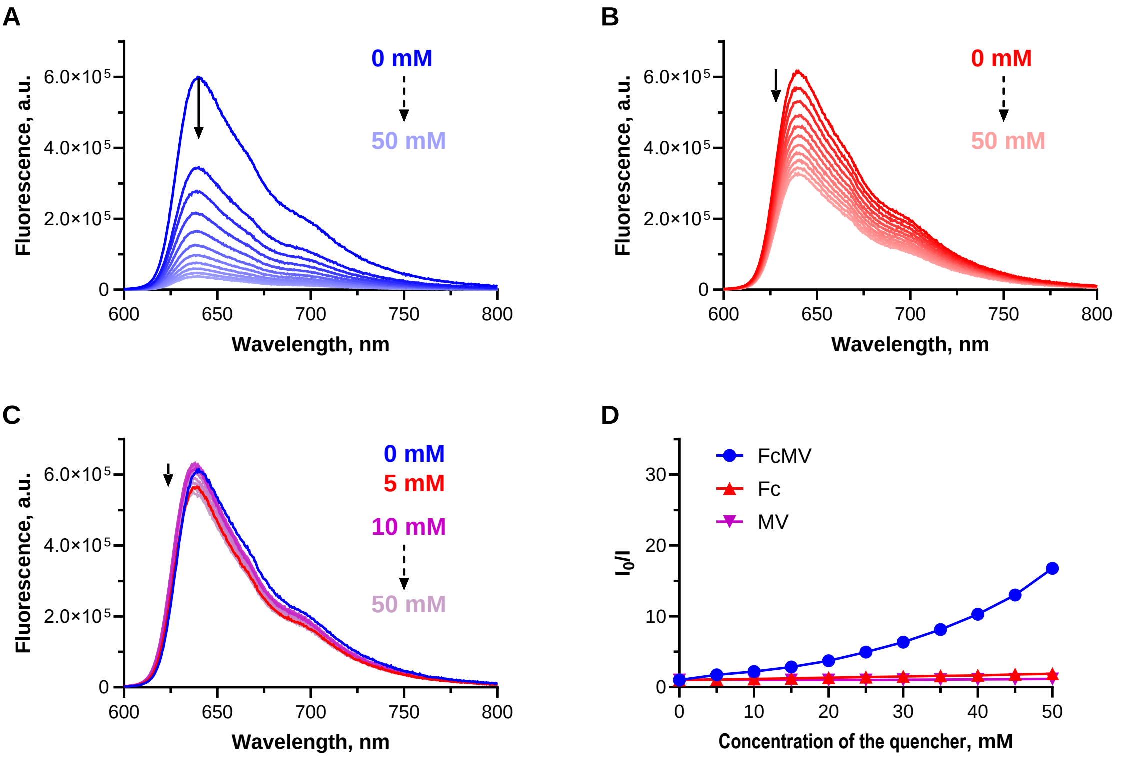

Figure S25. Fluorescence spectra of 5 µM solution of **2** in MeCN solutions of (A) **FcMV**, (B) **Fc** and (C) **MV** of different concentrations in the range of 0 mM to 50 mM, and (D) Stern-Volmer plots of AzaPc quenching by **FcMV** (blue), **Fc** (red) and **MV** (purple) calculated from the intensities of fluorescence emission at 646 nm (I_0 corresponds to the intensity of pure **1**; I is the intensity at the given concentration of the quencher; excitation wavelength was 590 nm).

2.2. Stern-Volmer plots for **Fc** and **MV**

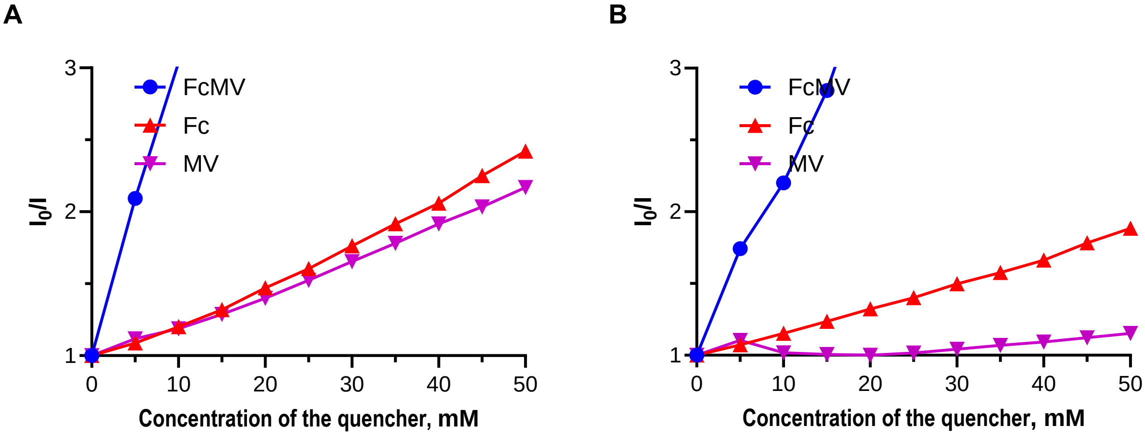

Figure S26. Details of Stern-Volmer plots of AzaPcs (A) **1** and (B) **2** quenching by **FcMV** (blue), **Fc** (red) and **MV** (purple) calculated from the intensities of fluorescence emission at 646 nm (I_0 corresponds to the intensity of pure AzaPc; I is the intensity at the given concentration of the quencher; excitation wavelength was 590 nm) – detail to see the

2.3. Lifetimes (AzaPc **1**)

Table S1. Lifetime components (τ_1 and τ_2 for biexponential and τ for monoexponential decay) in ns of **1** during the titration by **FcMV**, **Fc** and **MV**, and the relative contribution of both components.

	Concentration/M	Parameter	Value/ns	Rel.%
**FcMV**	0.000	τ	2.0940	100.00
	0.005	τ_1	0.3920	3.99
		τ_2	1.7902	96.01
	0.015	τ_1	0.2674	5.67
		τ_2	1.4873	94.33
	0.030	τ_1	0.2773	10.22
		τ_2	1.2308	89.78
	0.050	τ_1	0.2479	14.55
		τ_2	1.0013	85.45
**Fc**	0.000	τ	2.0879	100.00
	0.005	τ	1.8954	100.00
	0.015	τ	1.6260	100.00
	0.030	τ	1.3382	100.00
	0.050	τ	1.0730	100.00
**MV**	0.000	τ	2.0751	100.00
	0.005	τ_1	0.6101	4.90
		τ_2	2.2891	95.10
	0.015	τ_1	0.5539	6.67
		τ_2	2.2950	93.33
	0.030	τ_1	0.3295	6.76
		τ_2	2.1405	93.24
	0.050	τ_1	0.3153	10.28
		τ_2	2.0124	89.72

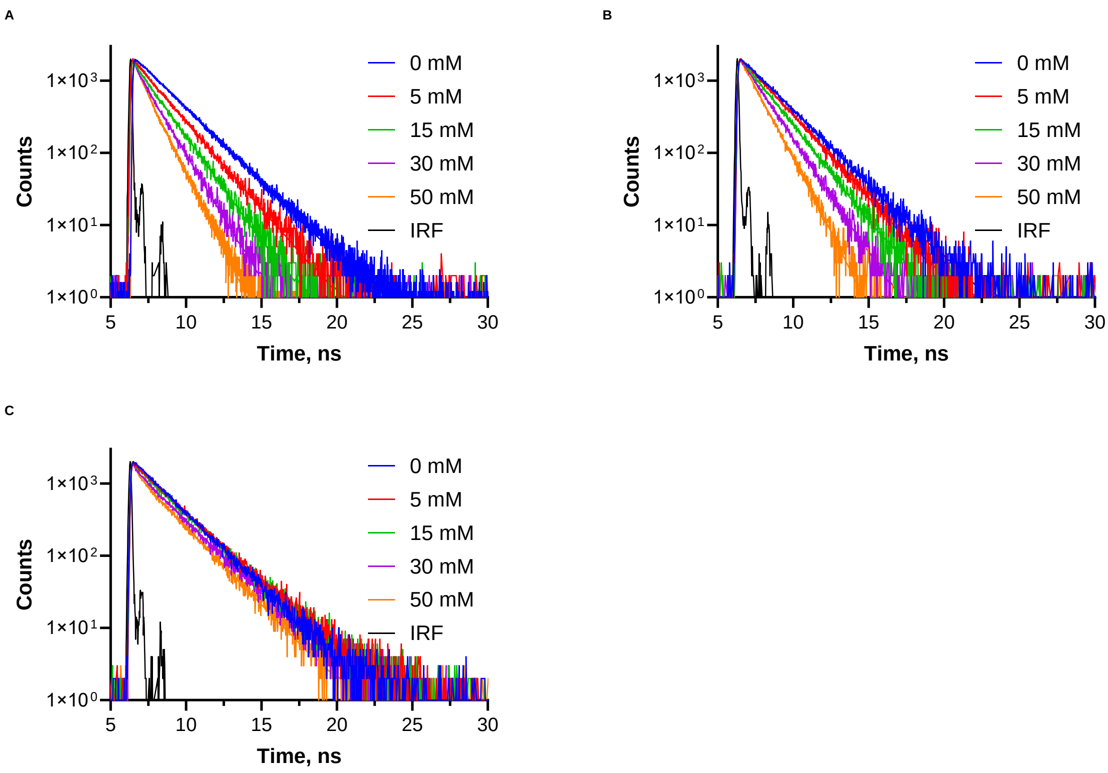

Figure S27. Fluorescence decays from the lifetime measurements during titration of **1** (5 µM, MeCN) with (A) **FcMV**, (B) **Fc** and (C) **MV** of different concentrations in the range of 0 mM to 50 mM.

2.4. Calculations

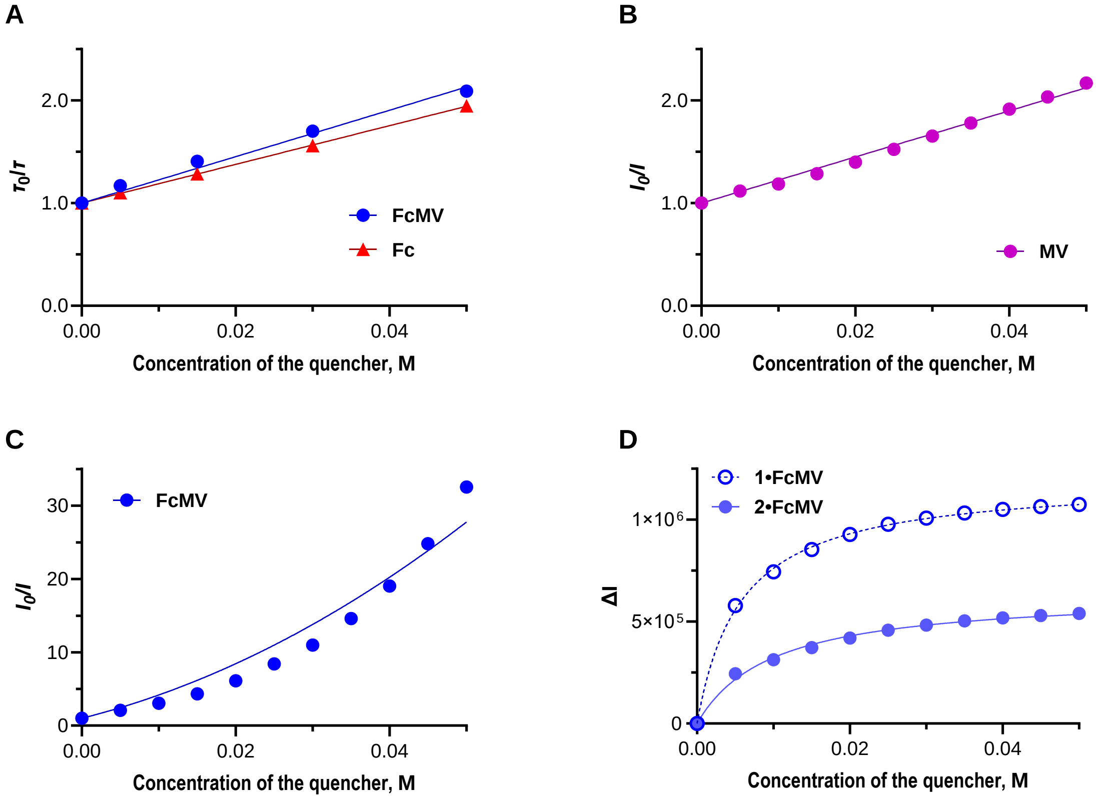

Figure S28. Regression curves fitted according to Equations 1 to 6. Linear regression for calculations of (A) K_D of **FcMV** and **Fc**, and (B) K_S of **MV**. Non-linear regression of for calculations of (C) K_S of **FcMV**. (D) Regression curve fitted according to Equation 6 for the calculation of K_a of the complex of **FcMV** and AzaPc **1** (dashed line) or **2** (solid line).

3. TLCs

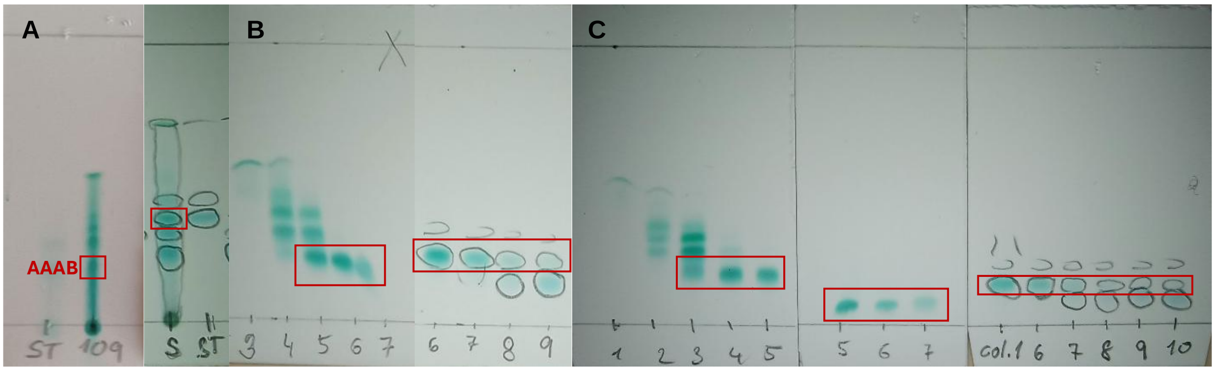

Figure S29. Analytical TLCs of (A) two examples of the reaction mixture from synthesis of unsymmetrical AzaPc **6** (mobile phase: DCM/MeOH/pyridine, 290 : 30 : 3, v/v/v), (B) fraction from the first column (mobile phase: DCM/MeOH/pyridine, 195 : 5 : 2, v/v/v), and (C) Fractions from the second column (purification of impure fractions from first column; mobile phase: DCM/MeOH/pyridine, 195 : 5 : 2, v/v/v).

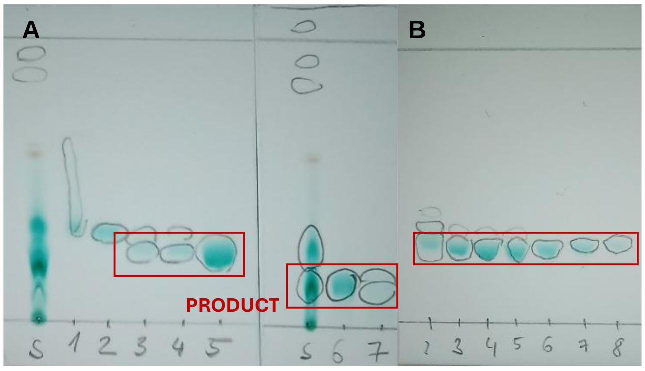

Figure S30. Analytical TLCs of the reaction mixture (labeled as S) and fractions during (A) first and (B) second column during the synthesis of AzaPc **1** by click-reaction.

4. Electrochemistry

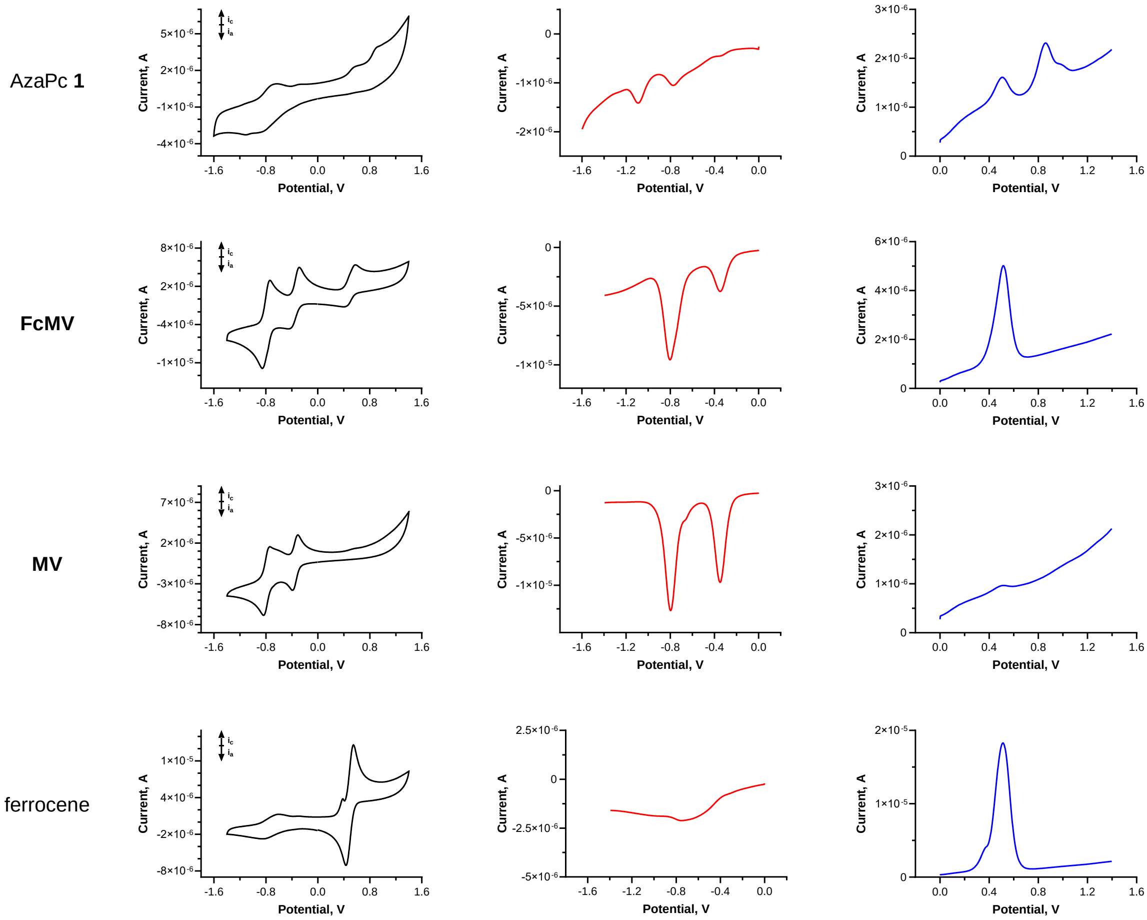

Figure S31. Cyclic voltammograms (left, black) and square-wave voltammograms (reduction – middle, red; oxidation – right, blue) in pyridine (100 mV/s, 0.1 M tetrabutylammonium hexafluorophosphate as supporting electrolyte, 25 °C) of AzaPc **1**, **FcMV**, **MV** and ferrocene. Potential vs. SCE was determined according to oxidation of ferrocene used as internal standard, E(Fc/Fc$^+$) = 0.56 V vs. SCE,[2] solutions of presented voltammograms do not contain ferrocene.

5. Transient Absorption Spectroscopy

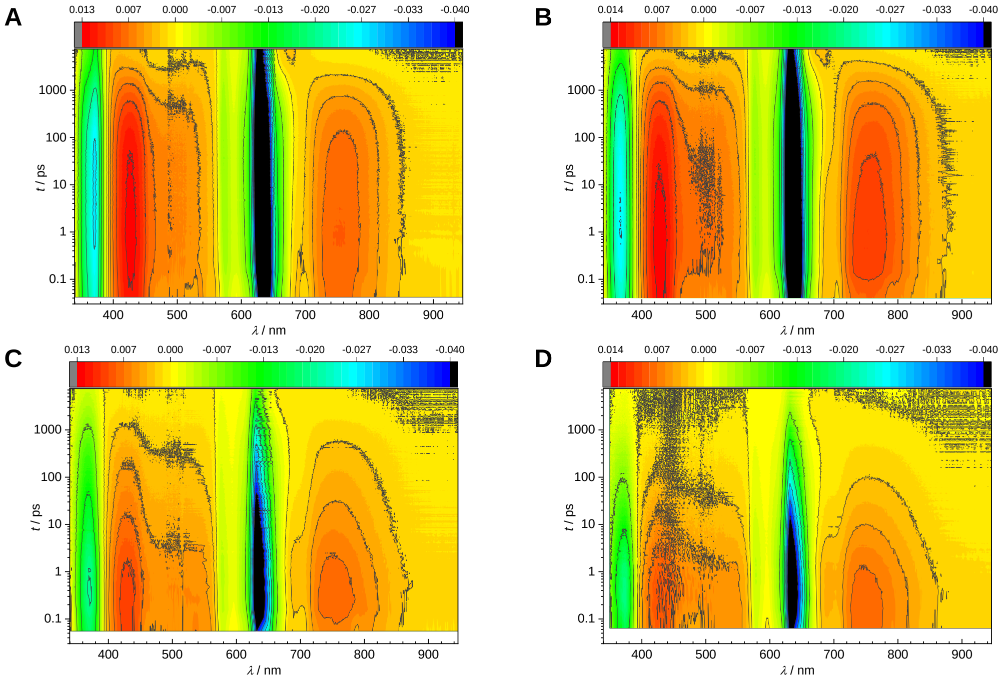

Figure S32. Picosecond TAS data of AzaPc **2** (A, 30 µM), AzaPc_**1** (B, 30 µM), AzaPc_**1** with **FcMV** (C, 15 µM and 12.5 mM, respectively), AzaPc_**1** with **FcMV** (D, 30 µM and 50 mM, respectively) in MeCN. Samples were excited at 640 nm and 200 nJ pump energy. Data are corrected for coherent artifacts.

6. References

(1) MSTools. *Analyse molecular formula - Isotopic distribution and elemental analysis*. https://mstools.epfl.ch/info/ (accessed 30. 10. 2025).

(2) Connelly, N. G.; Geiger, W. E. Chemical Redox Agents for Organometallic Chemistry. *Chemical Reviews* **1996**, *96* (2), 877-910. DOI: 10.1021/cr940053x.
